# A pH‐Responsive fluorescence lifetime probe reveals GOT1 phase separation in cellular anti‐senescence

**DOI:** 10.1002/smo2.70096

**Published:** 2026-09-03

**Authors:** Ziyue Chen, Jiaqi Li, Yan Bao, Mengmeng Wang, Haoyan Chen, Mingjiong Zhang, Lina Ding, Shuangshuang Wu, Baoxing Shen

**Affiliations:** ^1^ State Key Laboratory of Microbial Technology Nanjing Normal University Nanjing China; ^2^ School of Food Science and Pharmaceutical Engineering Nanjing Normal University Nanjing China; ^3^ School of Pharmaceutical Sciences Zhengzhou University Zhengzhou China; ^4^ Jiangsu Provincial Key Laboratory of Geriatrics Department of Geriatrics The First Affiliated Hospital of Nanjing Medical University Nanjing China

**Keywords:** anti‐senescence, fluorescence lifetime imaging, GOT1 liquid‐liquid phase separation, pH probe

## Abstract

Cellular senescence involves progressive acidification, but how cells sense and adapt to this pH shift remains unclear. Here we report that metabolic enzyme GOT1 functions as a pH sensor that undergoes liquid–liquid phase separation (LLPS) to combat senescence. Proteomic analysis identified GOT1 upregulation in aged human lung cells. Acidic conditions mimicking senescence directly induce GOT1 LLPS via its N‐terminal intrinsically disordered region (IDR1), recruiting ME1 to form dynamic enzymatic co‐condensates that scavenge reactive oxygen species and alleviate oxidative stress. To quantitatively interrogate GOT1's pH microenvironment during senescence, we engineered BDP‐PLP, a first‐in‐class fluorescent probe conjugating the native GOT1 cofactor pyridoxal phosphate to a BODIPY fluorophore. Operating via a binding‐inhibited PET mechanism, this probe enables high‐specificity GOT1 targeting and pH‐dependent fluorescence lifetime imaging (FLIM). Using FLIM, we achieved quantitative real‐time visualization of pH dynamics within GOT1 condensates in living cells, revealing that phase separation generates a highly acidic local microenvironment critical for its anti‐senescence function. This study uncovers a pH‐triggered phase separation mechanism that bolsters antioxidant defense via metabolic enzyme co‐condensation, offering new perspectives on metabolic adaptation in aging and establishing a chemical tool for probing microenvironmental dynamics.

## Graphical abstract

Acidic pH triggers GOT1 phase separation via IDR1, recruiting ME1 to form ROS‐scavenging condensates that combat senescence, revealed by a FLIM‐based pH probe.

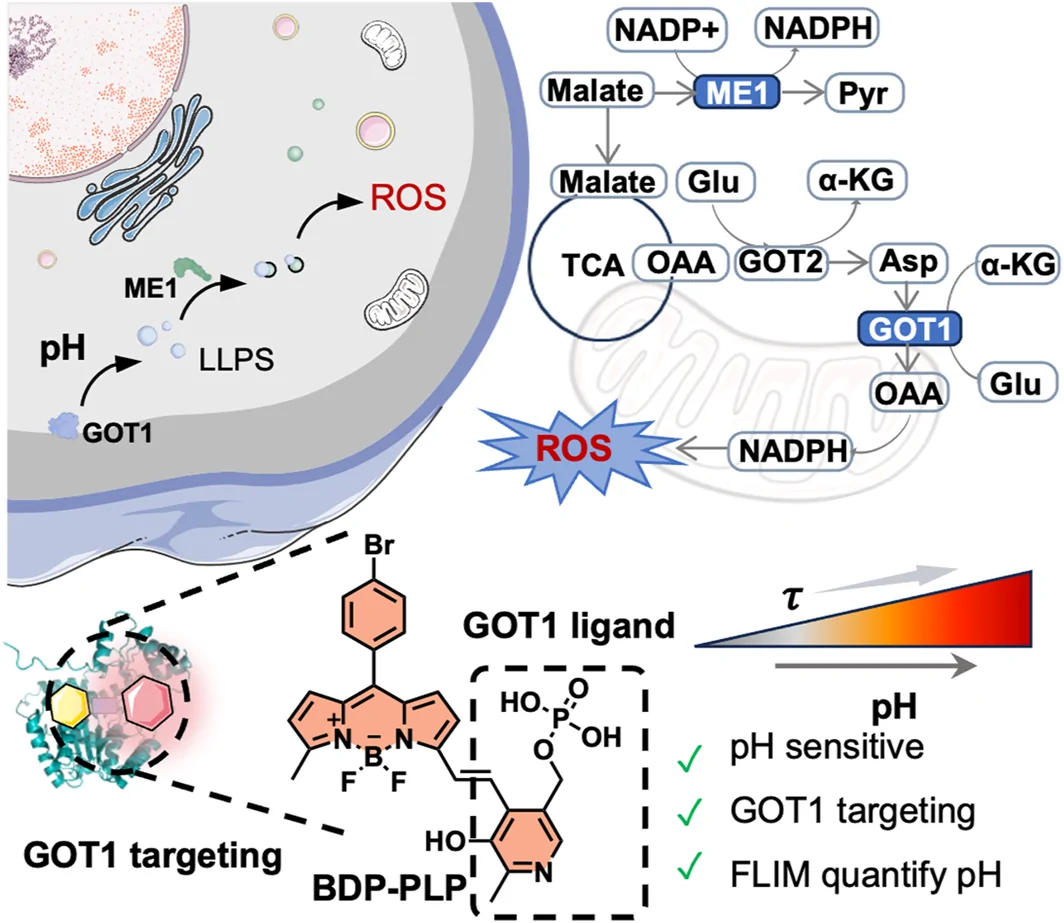

## 1 INTRODUCTION

Senescence represents a stable state of proliferative arrest that is intimately linked to organismal aging and the progression of age‐related pathologies.[^1^, ^2^] In addition to the well‐established hallmarks of cell cycle exit and the senescence associated secretory phenotype (SASP),^[3]^ senescent cells undergo profound alterations in metabolic reprogramming and ion homeostasis.[^4^, ^5^] Recent evidence highlights the critical importance of intracellular pH stability for normal cellular function^[6]^; notably, senescent cells frequently exhibit a more acidic cytoplasmic milieu.[^7^, ^8^] Whether this acidic shift is merely a passive consequence of senescence or serves as an active signaling cue that shapes senescent phenotypes and cellular fate remains unknown. Understanding how cells perceive acid stress and convert it into adaptive responses is therefore a pivotal step toward deciphering the biology of senescence.

In recent years, liquid‐liquid phase separation (LLPS) has emerged as a fundamental physical mechanism driving the assembly of biomolecules into membrane less organelles,[^9^, ^10^] governing diverse biological processes such as gene expression, signal transduction, and cellular stress adaptation.[^11^, ^12^, ^13^, ^14^] Studies indicate that numerous metabolic enzymes can undergo phase separation under specific physiological or stress conditions, thereby enabling precise spatiotemporal regulation of intracellular metabolism.^[15]^ These findings suggest that phase separation may represent a key strategy through which cells sense microenvironmental changes and reprogram their metabolic states.[^16^, ^17^] Nevertheless, it remains to be investigated whether metabolic sensors exist in the acidic environment associated with cellular senescence that regulate the cell's growth state through specific molecular mechanisms.

**Scheme 1:**
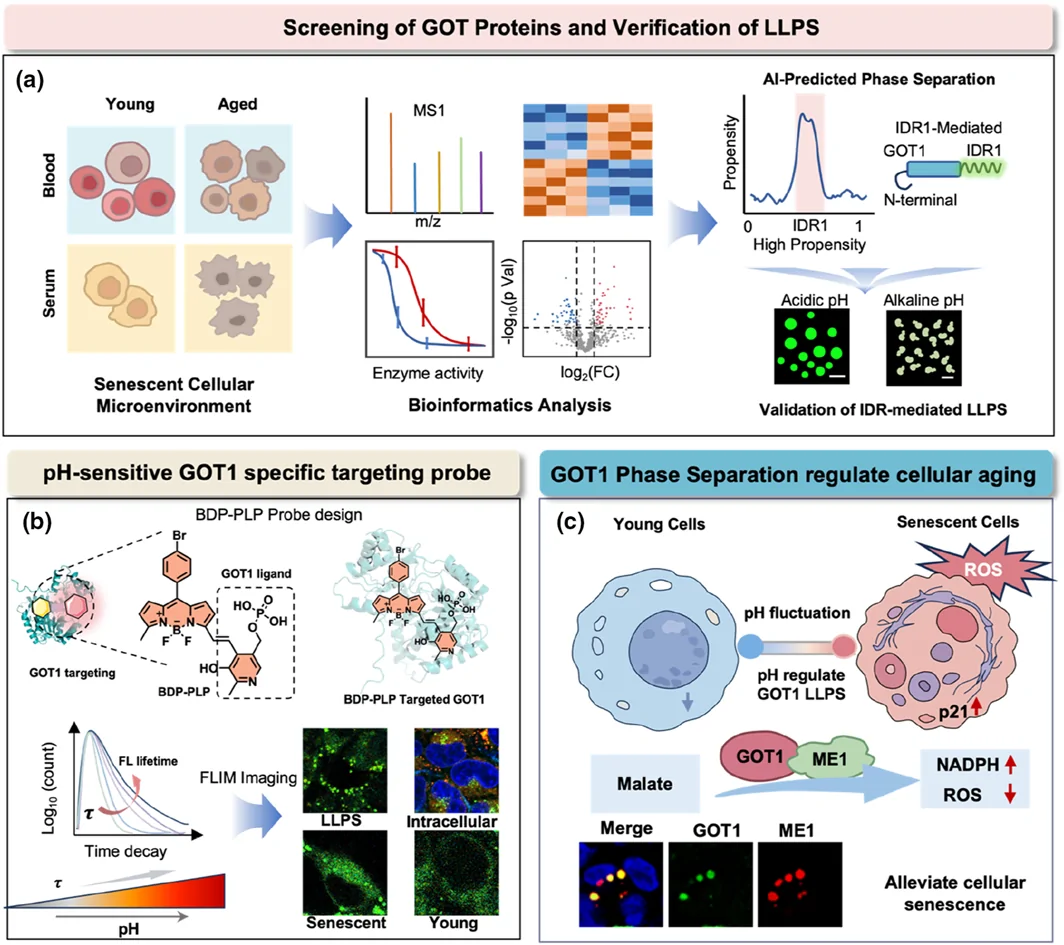
Validation of GOT1 phase separation mechanism and probe design. (a) Proteomics analysis of differentially expressed proteins and phase separation validation. Differentially expressed proteins were identified through database searches and bioinformatics analysis, with phase separation mechanisms validated via qPCR and Western blotting. (b) Design of pH‐responsive fluorescent probes; Fluorescent probes labeled with GOT1‐specific ligands for intracellular imaging; Effects of phase separation on biological mechanisms and quantitative intracellular pH analysis via fluorescence lifetime imaging (FLIM). (c) Cytoplasmic pH decline induced by cellular senescence triggers GOT1 phase separation. Its interaction with ME leads to reduced expression of the senescence marker p21 and decreased ROS levels.

Through quantitative proteomic profiling, this study identified glutamate oxaloacetate transaminase 1 (GOT1) as markedly upregulated in senescent cells (Scheme 1a). As a central enzyme in the malate aspartate shuttle, GOT1 bridges cytoplasmic and mitochondrial metabolic networks.[^18^, ^19^] Subsequent experiments demonstrated that an acidosis mimicking environment, reminiscent of the senescent niche, directly triggers liquid‐liquid phase separation (LLPS) of GOT1. This phase transition event correlated significantly with reduced levels of the senescence marker p21, suggesting that the phase transition of GOT1 may participate in regulating senescence associated biological processes.

To elucidate the underlying mechanism, we further showed that GOT1 specifically interacts with malic enzyme 1 (ME1),^[20]^ a critical metabolic node connecting glycolysis to nicotinamide adenine dinucleotide phosphate hydrogen (NADPH) generation. Under acidic conditions, GOT1 and ME1 undergo cooperative phase separation, forming multic enzyme condensates. Functional assays confirmed that these condensates effectively lower intracellular reactive oxygen species (ROS) levels, thereby mitigating oxidative stress and furnishing senescent cells with a survival adaptive mechanism.

Furthermore, to precisely quantify the dynamic changes in intracellular pH during aging and establish a causal relationship with the aforementioned mechanism,^[21]^ we developed a novel pH‐responsive fluorescence lifetime probe (BDP‐PLP) targeting the GOT1 protein (Scheme 1b). In intracellular pH monitoring, traditional fluorescence‐based probes are susceptible to interference from factors such as probe concentration, excitation light intensity, and photobleaching, making it difficult to achieve long‐term stable quantitative measurements.[^22^, ^23^, ^24^, ^25^, ^26^, ^27^] Fluorescence lifetime, as a steady‐state photophysical parameter, offers unique advantages including independence from probe concentration and excitation intensity, high resistance to photobleaching, superior resolution, and quantitative analytical capability, making it ideal for high‐precision measurement of microenvironmental pH within living cells.[^28^, ^29^, ^30^, ^31^, ^32^, ^33^] In recent years, FLIM‐based quantitative analysis methods have been widely used to assess the degree of aging in cells and organisms in vivo.[^34^, ^35^] This demonstrates that FLIM, when combined with targeted pH‐sensitive probes, is a powerful tool for studying dynamic changes in the intracellular microenvironment. In this study, by integrating fluorescence lifetime imaging microscopy (FLIM), we quantitatively analyzed the dynamic changes in local microenvironmental pH during the phase transition of the GOT1 inside live cells.

In this study, we tested the hypothesis that deviation in intracellular pH serves as a mechanism to guide the regulation of cellular senescence. We found that senescent cells exhibit a global acidification trend, which induces GOT1 protein phase separation and recruits the metabolic enzyme ME1 to form co‐condensates. This phase separation process significantly reduces intracellular reactive oxygen species (ROS) levels and enhances antioxidant capacity, thereby mitigating cellular senescence (Scheme 1c). Additionally, by manipulating the ability of GOT1 to undergo phase separation, we observed corresponding alterations in cellular senescence phenotypes, further confirming the functional role of GOT1 condensation in senescence regulation. This study provides a novel perspective for understanding how senescent cells maintain homeostasis.

## 2 RESULTS AND DISCUSSION

### 2.1 Identification of GOT1 via proteomic and bioinformatic screening

**FIGURE 1:**
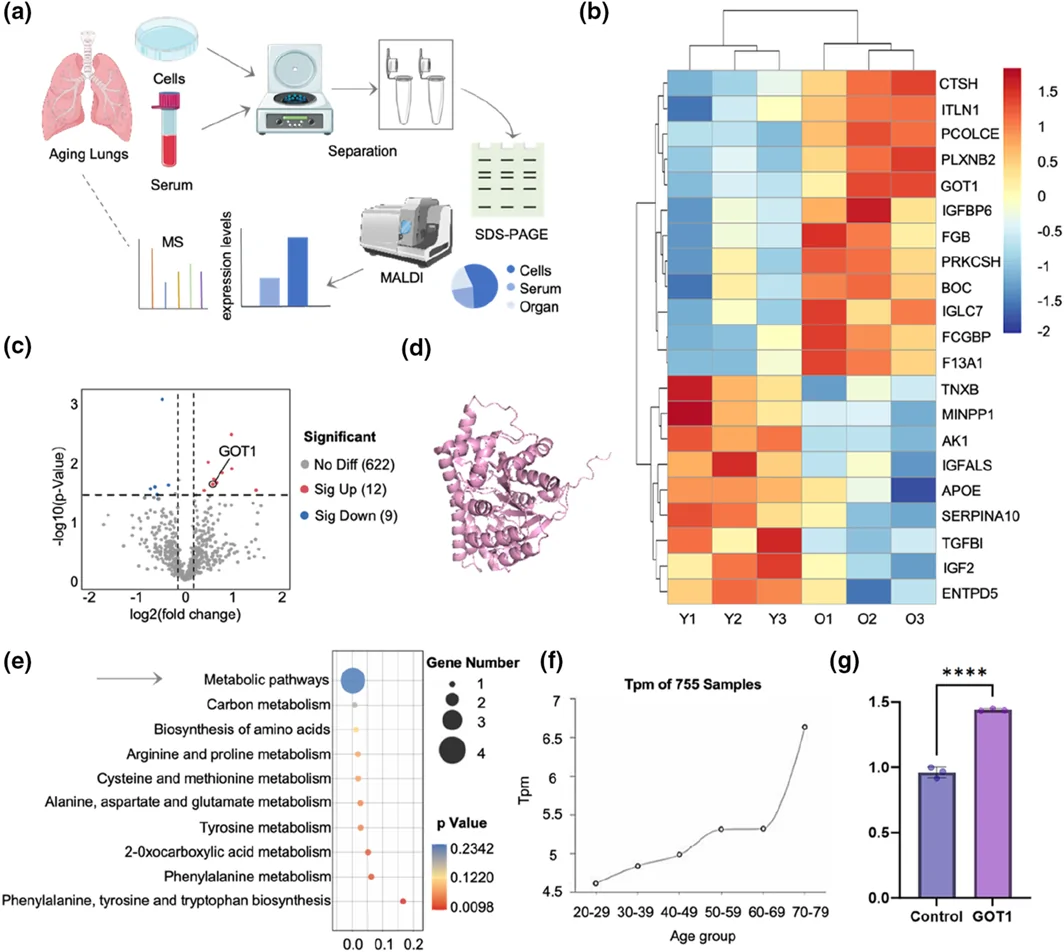
Proteomics and bioinformatics analysis. (a). Schematic diagram of the proteomics analysis experimental design, involving proteomics analysis of peripheral blood cells and serum samples from young and aged individuals (strictly age‐matched). (b). Cluster analysis of highly expressed proteins in serum. The heatmap displays proteins exhibiting consistent expression changes across different donor sera. Rows represent proteins, columns represent samples (Y denotes young group; O denotes old group). The color gradient indicates normalized protein expression levels. (c) Age‐related differentially expressed proteins. Volcano plot showing the statistical distribution of all differentially expressed proteins across samples. Each point represents one protein; the *X*‐axis denotes fold change (Log_2_ fold change), and the *Y*‐axis denotes statistical significance (−Log_10_ *p*‐value). GOT1 expression is significantly upregulated. Gray dashed lines mark the significance threshold (*p*‐value <0.05, fold change >1.5). (d) Three‐dimensional structural model of the GOT1 protein. (e) KEGG pathway enrichment analysis. The bubble plot displays KEGG signaling pathways significantly enriched in altered proteins from aged samples, sorted by enrichment factor. Alanine, aspartate, and glutamate metabolism pathways are significantly enriched. (f) Correlation between GOT1 transcriptional expression and donor age in the GTE x database. The scatter plot displays data from 755 human lung tissue samples sourced from the Genotype‐Tissue Expression (GTE x) database. GOT1 gene expression levels (transcripts per million reads, TPM) showed a significant positive correlation with donor age (years). The figure indicates Pearson correlation coefficients (*r*) and corresponding *p*‐values. (g) qPCR validation of GOT1 mRNA upregulation in cellular senescence models. GOT1 mRNA expression levels were detected via qPCR in human lung epithelial cells (BEAS‐2B) undergoing senescence. The bar chart shows significantly elevated GOT1 transcription levels in senescent cells compared with young proliferating cells (*n* = 3, *p* < 0.0001). Primer information for GOT1 and GAPDH is provided in the table in the appendix (Supporting Information S1 Table S5).

To systematically reveal key molecular drivers in aging, we performed comprehensive quantitative proteomics analysis on peripheral blood cells and serum samples from young and aged donors with strict age matching (Figure 1a, Supporting Information S1 Table S3). Initially, via cluster analysis of high‐resolution mass spectrometry (HRMS) data, we identified proteins with consistent upregulation in the serum of elderly subjects (Figure 1b). Subsequent statistical screening of differentially expressed proteins (DEPs) through volcano plot analysis further validated that mitochondrial aspartate aminotransferase (GOT1) was among the most significantly upregulated proteins in aged samples (Figure 1c).[^36^, ^37^] To elucidate its potential function from a structural perspective, we obtained the crystal structure of human GOT1 protein (PDB ID: 6DND) from the PDB database and utilized PyMOL software for three‐dimensional structural visualization analysis and functional site investigation (Figure 1d).

To further dissect the biological implications of GOT1 upregulation, KEGG pathway enrichment analysis was conducted on all aging‐associated differentially expressed proteins (DEPs). These analyses revealed significant enrichment in metabolic pathways (most notably alanine, aspartate, and glutamate metabolism) as well as signaling pathways related to cellular senescence and autophagy (Figure 1e). To validate this observation at the cellular level and investigate the role of GOT1 overexpression in vivo, we analyzed RNA‐seq data from 755 human lung tissue samples in the integrating platform of age‐dependent expression and immune profiles across human tissues (ADEIP) database. The results demonstrated a significant positive correlation between the transcriptional levels of GOT1 (quantified as transcripts per million, TPM) and donor age (Figure 1f). Additionally, we analyzed differentially expressed genes in the serum transcriptome (Supporting Information S1 Figure S1, Table S2), confirming its transcriptional association with aging. To further investigate the association between GOT1 upregulation and cellular senescence at the functional level, we conducted proteomic profiling of peripheral blood cells (Tables S1 and S3; Supporting Information S1 Figures S2 and S3). Interestingly, no consistent senescence‐associated proteomic changes were observed in circulating immune cells, suggesting that these cells may represent secretory derivatives originating from tissue‐resident senescent cells rather than autonomous participants in the senescent phenotype.

Lastly, to verify alterations in GOT1 expression in a functionally relevant cellular model, a senescence model was established using human bronchial epithelial (BEAS‐2B) cells (Supporting Information S1 Figures S2 and S3), with precise validation performed via quantitative real‐time polymerase chain reaction (qPCR). The results showed a significant upregulation of GOT1 mRNA expression in senescent cells relative to young proliferating cells (Figure 1g). Collectively, through proteomic screening, bioinformatic database analyses, and in vitro cellular model validation, we demonstrate that GOT1 expression is upregulated during human lung aging processes, thereby laying a robust molecular foundation for subsequent functional and mechanistic investigations.

### 2.2 Phase separation mechanism and pH dependence of GOT1 in vitro and in cellular

**FIGURE 2:**
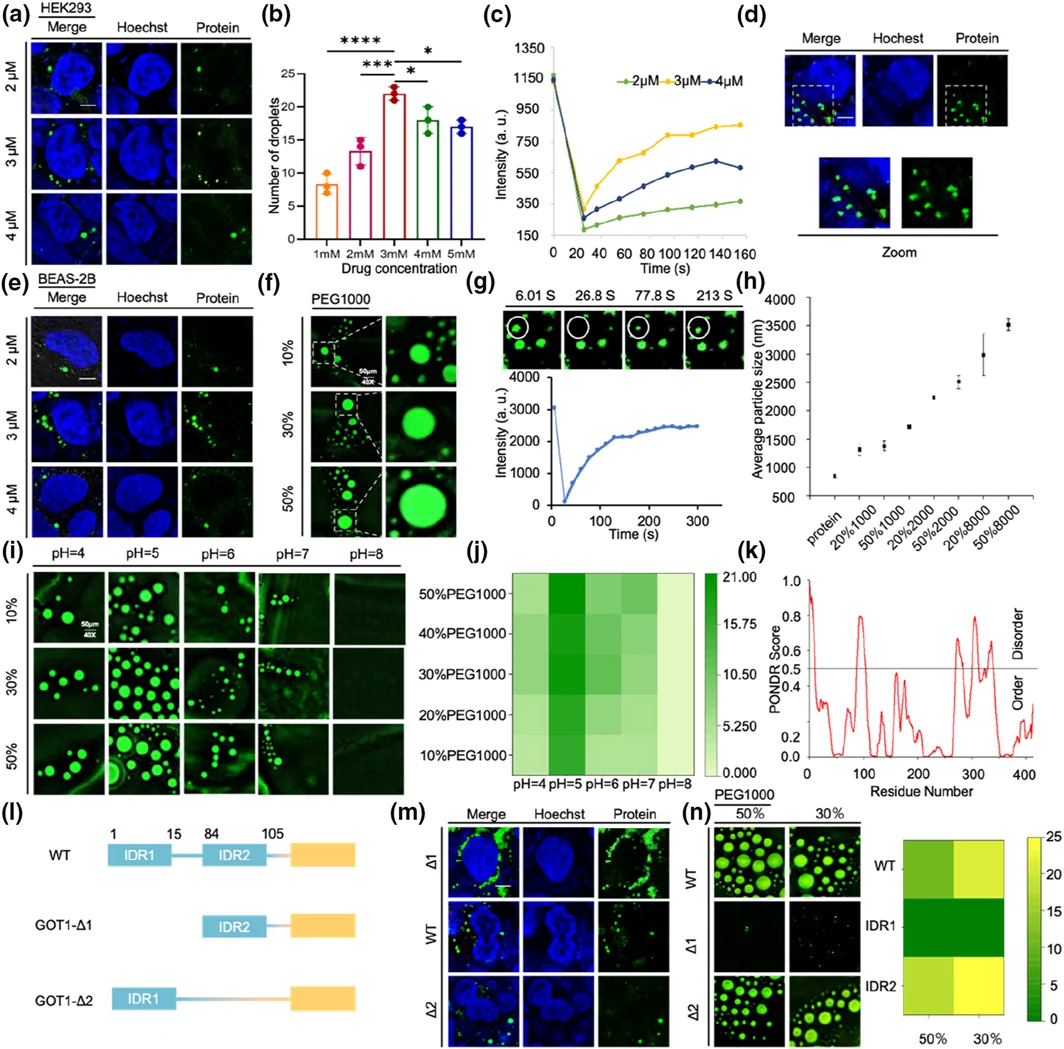
pH‐dependent phase separation of GOT1. (a) Overexpression of GOT1‐GFP in HEK293 cells. After 2 h of acid stress treatment, distinct spherical fluorescent condensates formed. Scale bar: 10 μm. (b) Quantitative analysis of GOT1 aggregate numbers in cells treated with different pH buffers (*n* = 3). At least 10 cells analyzed per condition. (c) Photobleaching recovery experiment. (d) Intracellular condensation state after 1,6‐hexanediol (1,6‐HD) treatment. (e) Replicated intracellular phase separation experiment in human lung epithelial cells (BEAS‐2B), scale bar: 10 μm. (f) In vitro phase separation experiment with purified wild‐type (WT) GOT1. Different concentrations of the crowding agent PEG‐1000 (10%, 30%, 50%) induced GOT1 droplet formation in PBS buffer (pH 7.4). Scale bar: 50 μm. (g) Fluorescence recovery after photobleaching (FRAP) analysis images (top) and photobleaching recovery curves (bottom) of GOT1 droplets formed in vitro. (h) Dynamic light scattering (DLS) analysis of protein droplet size changes in solution induced by varying PEG concentrations. (i) Effect of pH on GOT1 phase separation in vitro at a fixed PEG‐1000 concentration (30%). Scale bar: 50 μm. (j) Heatmap of GOT1 at different protein concentrations under various pH conditions. (k) IUPred2 software prediction of the GOT1 amino acid sequence identified two potential intrinsically disordered regions (IDRs): IDR1 and IDR2 (PONDR score >0.7). (l) Schematic diagram of the GOT1 protein domains, labeling the positions of IDR1 (amino acids 1–15) and IDR2 (amino acids 84–106). (m) Intracellular expression of wild‐type WT GOT1, IDR1 deletion mutant (ΔIDR1), and IDR2 deletion mutant (ΔIDR2). Acid stress‐induced condensate formation was observed only in the WT and ΔIDR2 groups, while ΔIDR1 lacked this ability. Scale bar: 10 μm. (n) In vitro phase separation assay showing droplet formation of purified WT and knockout ΔIDR1, ΔIDR2 GOT1 under 30% PEG‐1000 and pH 5.0 conditions. Scale bar: 50 μm.

We first investigated whether GOT1 forms biomolecular condensates under physiological stress conditions. Live‐cell imaging revealed that GOT1‐GFP, which exhibited a diffuse cytoplasmic distribution under basal conditions, assembled into spherical, highly fluorescent puncta upon intracellular pH modulation using N‐acetyl‐L‐glutamine (Figure 2a). Quantitative analysis demonstrated that acidification markedly enhanced GOT1 condensate formation in a dose‐dependent manner (Figure 2b).

To characterize the biophysical nature of these structures, we performed fluorescence recovery after photobleaching (FRAP) assays. GOT1‐GFP condensates displayed robust fluorescence recovery (∼70%) following photobleaching under optimal stimulating conditions (3 μM), indicative of the rapid molecular exchange characteristic of liquid‐like droplets (Figure 2c). Consistent with liquid‐liquid phase separation (LLPS) mediated by weak multivalent interactions, treatment with 1,6‐hexanediol disrupted hydrophobic interactions, abolished condensate formation and prevented fluorescence recovery (Figure 2d). To assess the generality of this phenomenon across cell types, we extended our analysis to human bronchial epithelial cells (BEAS‐2B). Lactate‐induced intracellular acidification similarly drove GOT1 phase separation in these cells (Figure 2e), suggesting that pH‐dependent GOT1 condensation is a conserved cellular response.

To dissect the physicochemical determinants of GOT1 phase separation under defined biochemical conditions, we recombinantly expressed and purified His‐tagged wild‐type GOT1 from E. coli (Supporting Information S1 Figure S4). In vitro reconstitution experiments using a molecular crowding agent (PEG 1000) revealed that purified GOT1 spontaneously formed spherical droplets (Figure 2f, Supporting Information S1 Figure S5). These in vitro droplets exhibited rapid fluorescence recovery following photobleaching, confirming their liquid‐like properties (Figure 2g). Systematic titration revealed that the GOT1 phase separation capacity peaked at pH 5.0 (Figure 2h). Construction of a phase diagram from turbidity measurements delineated the concentration‐ and pH‐dependent boundaries of GOT1 LLPS (Figure 2i).^[38]^ Dynamic light scattering (DLS) analysis further demonstrated a progressive increase in the hydrodynamic radius of GOT1 assemblies with increasing PEG concentration (Figure 2j), consistent with condensate maturation.

To identify the structural determinants driving GOT1 phase separation, we employed computational prediction algorithms, which identified two putative intrinsically disordered regions (IDRs) located at the N‐terminus (IDR1) and C‐terminus (IDR2) of GOT1 (Figure 2k,l). To functionally validate these predictions, we generated deletion mutants lacking either IDR1 (ΔIDR1) or IDR2 (ΔIDR2). Strikingly, the ΔIDR1 mutant completely failed to form condensates both in cells and in vitro under low‐pH conditions (Figure 2m,n), whereas the ΔIDR2 mutant retained partial phase separation capacity. These results establish that the N‐terminal IDR1 is the primary driver of pH‐dependent GOT1 phase separation.

In order to investigate how IDR1 influences GOT1 phase separation, we analyzed IDR1, IDR2, and wild‐type GOT1 with ProtParam and peptide chemistry. We found that IDR1 is rich in acidic charge groups (Supporting Information S1 Figure S19a,c) and contains multiple ionizable residues whose protonation states are highly sensitive to pH changes. Meanwhile, the theoretical isoelectric point (pI) of full‐length GOT1 is approximately 6.785, while that of IDR1 is 3.742 (Supporting Information S1 Figure S19b,d), indicating that at pH values below this threshold, this region carries a net positive charge. Protonation of acidic residues reduces the net negative charge, while basic residues retain a positive charge, altering the electrostatic balance and promoting intermolecular interactions, thereby leading to phase separation. Analysis of hydrophilicity and hydrophobicity using ProtParam shows that IDR1 has a value of 0.053 and IDR2 has a value of −0.455, suggesting that they may promote weak polyvalent interactions under acidic conditions.

### 2.3 Verification of GOT1 phase separation‐induced cellular senescence

Accumulating evidence from previous studies demonstrates that senescent cells are frequently associated with a global reduction in intracellular pH (pH < 7.2).^[39]^ Protein liquid‐liquid phase separation (LLPS) is highly sensitive to environmental cues, including pH, and plays a pivotal role in regulating cellular stress adaptation and homeostasis maintenance.[^40^, ^41^] This raises the possibility that GOT1 LLPS under acidic conditions may constitute not merely a physical state transition but rather an adaptive cellular response to senescence‐associated acidification stress.

**FIGURE 3:**
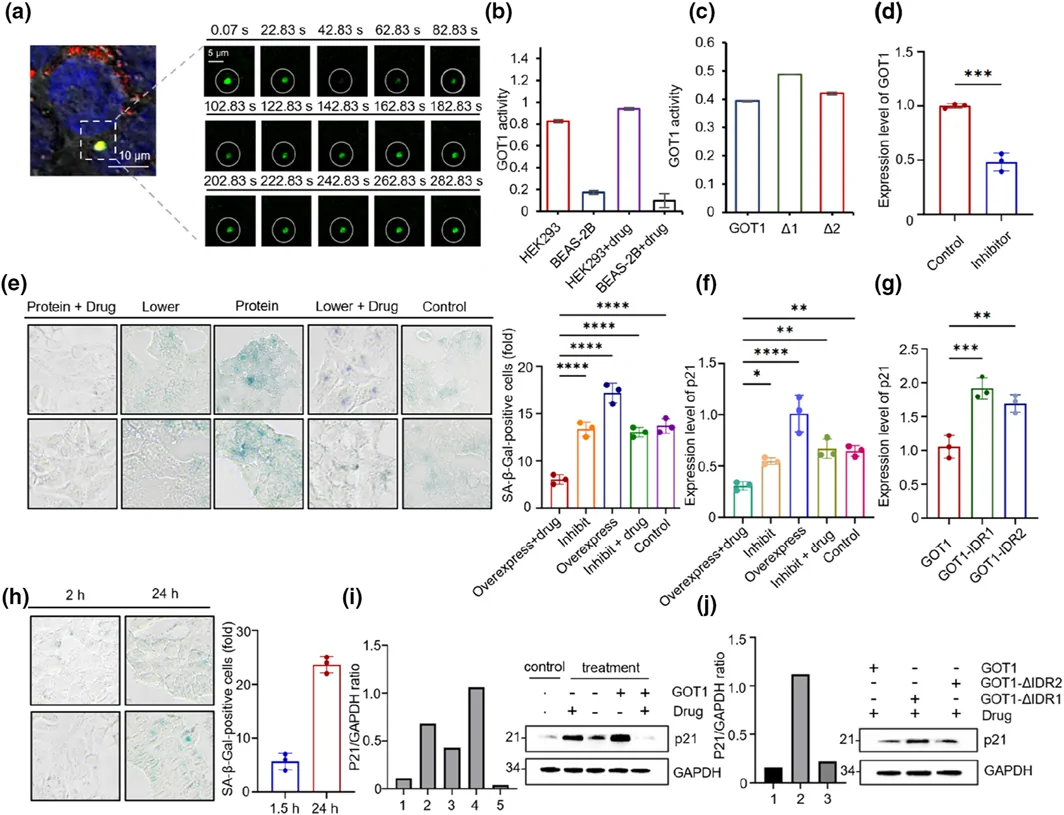
Functional validation of GOT1 phase separation. (a) Fluorescence recovery after photobleaching (FRAP) analysis images of GOT1 phase separation, demonstrating its dynamic liquid‐like properties. Scale bar, 10 μm. (b) Intracellular glutamate oxaloacetic transaminase (GOT) activity assays in HEK293 and BEAS‐2B cells before and after phase separation. (c) Intracellular GOT activity assays in HEK293 cells overexpressing wild‐type GOT1 (WT) or its phase separation‐deficient mutants (ΔIDR1, ΔIDR2) under GOT1 phase separation conditions. (d) qPCR experiments confirming mRNA expression levels of the GOT1 gene after GOT1 inhibitor treatment (*p* < 0.001). (e) Representative images of senescent *β*‐galactosidase (SA‐β‐gal) staining in HEK293 cells overexpressing GOT1 under GOT1 phase separation conditions (left) and quantitative analysis of the percentage of positive cells (right). (*p* < 0.0001). Scale bar, 20 μm. (*n* = 3, >50 cells per group). (f, i) Expression levels of the senescence marker p21 were detected by qPCR (f) and WB (i) in designated experimental groups (Nature control, GOT1 inhibition, GOT1 inhibition + drug, GOT1 overexpression, GOT1 overexpression + drug). (g, j) Detection of p21 expression in cells overexpressing wild‐type GOT1 (WT), phase separation‐deficient mutants (ΔIDR1, ΔIDR2), or drug‐treated cells via qPCR (g) and WB (j). (h) Representative senescence‐associated *β*‐galactosidase (SA‐β‐galactosidase) overexpression (OE) staining images (left) and quantitative analysis of positive cells (right) at different time points (2 h, 24 h) after drug treatment altering intracellular pH (*p* < 0.001). Scale bar: 20 μm (*n* = 3). Statistical significance was determined by *t*‐tests (d, f, g, i, j) (*p* < 0.05, *p* < 0.001).

To test this hypothesis, we further interrogated whether GOT1 LLPS exerts specific biological functions in the regulation of cellular senescence. We systematically interrogated the functional role of GOT1 LLPS during the cellular senescence process. First, we confirmed that drug‐induced GOT1 biomolecular condensates display typical liquid‐like properties. Fluorescence recovery after photobleaching (FRAP) assays revealed rapid fluorescence signal recovery in the bleached region over time (Figure 3a, Supporting Information S1 Figures S6 and S7), indicative of highly dynamic molecular exchange within GOT1 condensates, a hallmark of functional biomolecular condensates.

Having confirmed the liquid‐like physical properties of GOT1 condensates, we next focused on determining whether the classical enzymatic activity of GOT1 contributes to this process. As a key enzyme in the malate‐aspartate shuttle, GOT1 catalyzes the reversible transamination reaction between aspartate and *α*‐ketoglutarate.^[42]^ To rule out potential indirect effects of drug treatment or LLPS on the classical metabolic function of GOT1, we first quantified changes in enzyme activity during the reaction.

In both HEK293 and BEAS‐2B cells, drug treatment that induced LLPS did not alter total glutamic‐oxaloacetic transaminase (GOT) activity (Figure 3b). To further corroborate this finding, we performed parallel assays in cells overexpressing either wild‐type GOT1 or a phase‐separation‐deficient GOT1 mutant. Notably, despite effective induction of LLPS in wild‐type GOT1 by the tested drugs, aspartate transaminase activity in cell lysates remained unaltered (Figure 3c). Collectively, these results confirm that the senescence phenotypes observed subsequently are independent of the canonical metabolic catalytic activity of GOT1. Taken together, after confirming the physical properties of GOT1 condensates, we ruled out a direct contribution of GOT1's classical enzymatic activity to this process.

Subsequently, we established a causal relationship between GOT1 phase separation and senescence mitigation by means of targeted inhibition and overexpression of the GOT1 gene in cultured cells. Following successful downregulation of GOT1 protein expression using a specific inhibitor (Figure 3d), we systematically evaluated cellular senescence phenotypes. In cells with GOT1 overexpression and induced phase separation, the proportion of senescence‐associated *β*‐galactosidase (SA‐β‐gal)‐positive cells was significantly reduced (Figure 3e). Concomitantly, both the mRNA (Figure 3f) and protein levels (Figure 3i, Supporting Information S1 Figures S8 and S9) of p21, a key senescence marker, were markedly decreased. In contrast, GOT1 knockdown significantly exacerbated cellular senescence. Collectively, these data indicate that GOT1 overexpression alone is insufficient to exert anti‐aging effects; instead, its phase separation capacity is indispensable for this function.

To directly verify the essential role of phase separation in cellular aging, we performed a structure‐function analysis. Previous studies have demonstrated that the intrinsically disordered region (IDR) of the GOT1 protein is responsible for mediating its phase separation, and that a mutant lacking the first IDR segment (ΔIDR1) completely loses its phase separation ability. Functional assays showed that, compared with wild‐type GOT1 (which retains phase separation capability), cells expressing the ΔIDR1 mutant exhibited significantly elevated p21 expression at both the transcriptional and protein levels (Figure 3g,j and Supporting Information S1 Figure S9). These results strongly confirm that GOT1 maintains the normal physiological state of senescent cells strictly through its phase separation ability, rather than through other intrinsic protein properties.

Finally, to rule out the potential influence of the lactate drug itself on cellular senescence, we directly examined the immediate effect of intracellular pH disruption on senescence progression. By modulating intracellular pH, we found that short‐term (2‐h) treatment did not induce cellular senescence, whereas prolonged (24‐h) acidification significantly increased the number of SA‐β‐galactosidase‐positive cells (Figure 3h). This finding suggests that the persistent local acidic microenvironment formed by GOT1 phase separation may act as a key cellular regulatory signal that modulates senescence progression over extended time periods. In summary, GOT1 undergoes liquid‐liquid phase separation via its intrinsic disordered region, independent of its classical enzymatic activity. This phase separation is essential for the anti‐aging function of GOT1, and the associated local microenvironment acidification may represent a potential mechanism linking GOT1 phase separation to the regulation of cellular aging.

### 2.4 Acidic stress triggers cooperative liquid‐liquid phase separation of GOT1 and ME1

Cellular senescence is frequently accompanied by a reduction in intracellular pH. However, the molecular mechanisms by which cells sense and adapt to this acidic stress to sustain intracellular homeostasis remain poorly understood. The content of the above study demonstrated that GOT1 undergoes liquid‐liquid phase separation (LLPS) under acidic stress and exerts anti‐senescent effects, though the underlying molecular mechanisms remain elusive.

**FIGURE 4:**
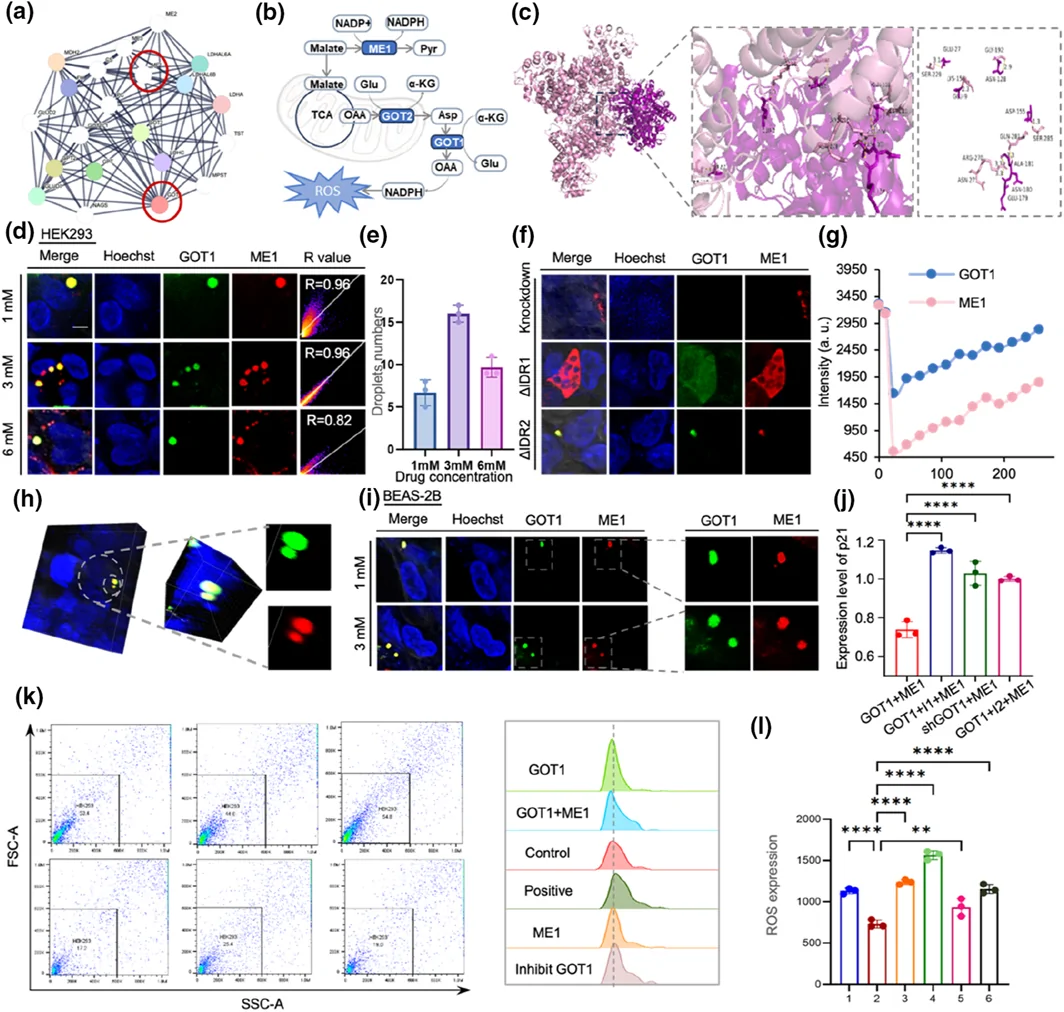
GOT1 and malic enzyme 1 (ME1) synergistically inhibit reactive oxygen species (ROS) production and regulate cellular senescence through phase separation. (a) Schematic model of the mechanism. In the acidic microenvironment associated with senescence, GOT1 interacts with ME1 and undergoes synergistic liquid‐liquid phase separation, forming a metabolic enzyme condensate. This inhibits ROS accumulation, thereby alleviating cellular senescence. (b) Bioinformatics prediction of the GOT1‐ME1 protein interaction network. (c) Molecular docking simulation results for GOT1 and ME1, showing complementary molecular surfaces and predicted binding interface. (d) Confocal microscopy image of HEK293 cells co‐expressing GOT1‐GFP (green) and ME1‐mCherry (red). Scale bar, 10 μm. Arrows indicate colocalized aggregates formed under acidic stress conditions. (e) Quantitative analysis of intracellular GOT1 and ME1 co‐precipitates under different pH conditions. (*n* = 3, *p* < 0.001). (f) Validation of GOT1's essentiality for ME1 phase separation. Control: cells treated with GOT1 inhibitor; Middle: cells expressing GOT1‐ΔIDR1 mutant; Right: cells expressing GOT1‐ΔIDR2 mutant. ME1‐mCherry failed to form aggregates under all conditions where GOT1 phase separation function was impaired. Scale bar, 10 μm. (g) Fluorescence recovery after photobleaching (FRAP) curves of GOT1 and ME1 co‐condensates demonstrate rapid fluorescence recovery. (h) Three‐dimensional surface reconstruction image of co‐condensates, revealing their three‐dimensional morphology. (i) Reproducibility validation of GOT1‐GFP and ME1‐mCherry colocalization in human lung epithelial cells (BEAS‐2B), confirming its universality. Scale bar, 10 μm. (j) qPCR analysis of the effect of GOT1 knockdown on the transcriptional level of the aging marker p21. (k) Flow cytometry detection of intracellular reactive oxygen species (ROS) levels. Blue peaks indicate cells undergoing GOT1 and ME1 co‐segregation under acidic stress, exhibiting left‐shifted (reduced) ROS signals. (l) Quantitative analysis of ROS levels (mean fluorescence intensity). Data are presented as mean ± standard deviation, *n* = 3 independent experiments. *p* < 0.001, *t*‐test significant.

To investigate whether GOT1 acts synergistically with other factors to mediate its anti‐senescent function, we performed bioinformatics analyses, which revealed strong predicted interactions between GOT1 and malate enzyme 1 (ME1). ME1 is a key metabolic enzyme that connects glycolysis to the malate‐aspartate shuttle and is responsible for the production of nicotinamide adenine dinucleotide phosphate (NADPH),^[43]^ a critical reducing agent (Figure 4a).

To confirm the physical interaction between GOT1 and ME1, we conducted molecular docking simulations. These simulations revealed high levels of electrostatic complementarity and favorable alignment of hydrophobic interfaces on the surfaces of the two proteins, supporting the formation of a stable GOT1‐ME1 complex (Figure 4b). Next, we co‐expressed GOT1 fused to green fluorescent protein (GOT1‐GFP) and ME1 fused to mCherry (ME1‐mCherry) in HEK293 cells. Confocal laser scanning microscopy (CLSM) imaging demonstrated that under physiological conditions, both proteins were diffusely distributed throughout the cytoplasm. Upon exposure to acidic stress, however, the two proteins rapidly colocalized and formed numerous spherical fluorescent puncta, indicative of condensates (Figure 4c).

This condensation phenomenon exhibited a pH‐dependent pattern, with the number of condensates increasing significantly as extracellular pH decreased (Figure 4d), and was strictly dependent on GOT1's LLPS capacity. When GOT1 expression was downregulated using a specific GOT1 inhibitor, or when its critical intrinsically disordered region (IDR) was ablated, ME1‐mCherry completely failed to form condensates, confirming that GOT1 is indispensable for this condensation process (Figure 4e).

Subsequently, we performed an in‐depth characterization of the physical properties of these GOT1‐ME1 co‐condensates. Fluorescence recovery after photobleaching (FRAP) assays demonstrated that the fluorescent signal within the condensates recovered rapidly following photobleaching, confirming the highly dynamic molecular exchange within these condensates, a hallmark of classical liquid‐phase condensates (Figure 4f). Three‐dimensional (3D) image reconstruction allowed us to visualize the spatial distribution and morphology of these condensates within the cytoplasm (Figure 4g). To verify the universality of this phenomenon, we repeated these experiments in human lung epithelial BEAS‐2B cells and observed highly consistent co‐condensation of GOT1 and ME1 (Figure 4h), indicating that the synergistic LLPS of GOT1 and ME1 is conserved across distinct cell types.

Finally, we investigated the functional consequences of the synergistic phase separation between GOT1 and ME1. Quantitative real‐time PCR analysis revealed that the formation of GOT1‐ME1 condensates significantly downregulated the transcriptional expression of p21 (Figure 4i). To dissect the structural determinants underlying this phase separation, we generated GOT1 deletion mutants lacking two distinct intrinsically disordered regions (IDRs). Overexpression of these variants showed that loss of the first IDR resulted in markedly higher p21 expression compared with the deletion of the second IDR, indicating a dominant role for this region in modulating senescence‐associated gene expression via phase separation. Notably, induction of GOT1‐ME1 phase separation robustly suppressed intracellular reactive oxygen species (ROS) accumulation (Figure 4j,k,l). Collectively, these findings demonstrate that acid stress promotes the recruitment of ME1 by GOT1 into dynamic metabolic condensates, which function as specialized hubs for efficient NADPH regeneration and ROS scavenging. This process disrupts the oxidative stress‐driven feedback loop associated with cellular senescence and thereby exerts a cytoprotective function.

### 2.5 GOT1‐targeting pH‐responsive fluorescence lifetime probe

**FIGURE 5:**
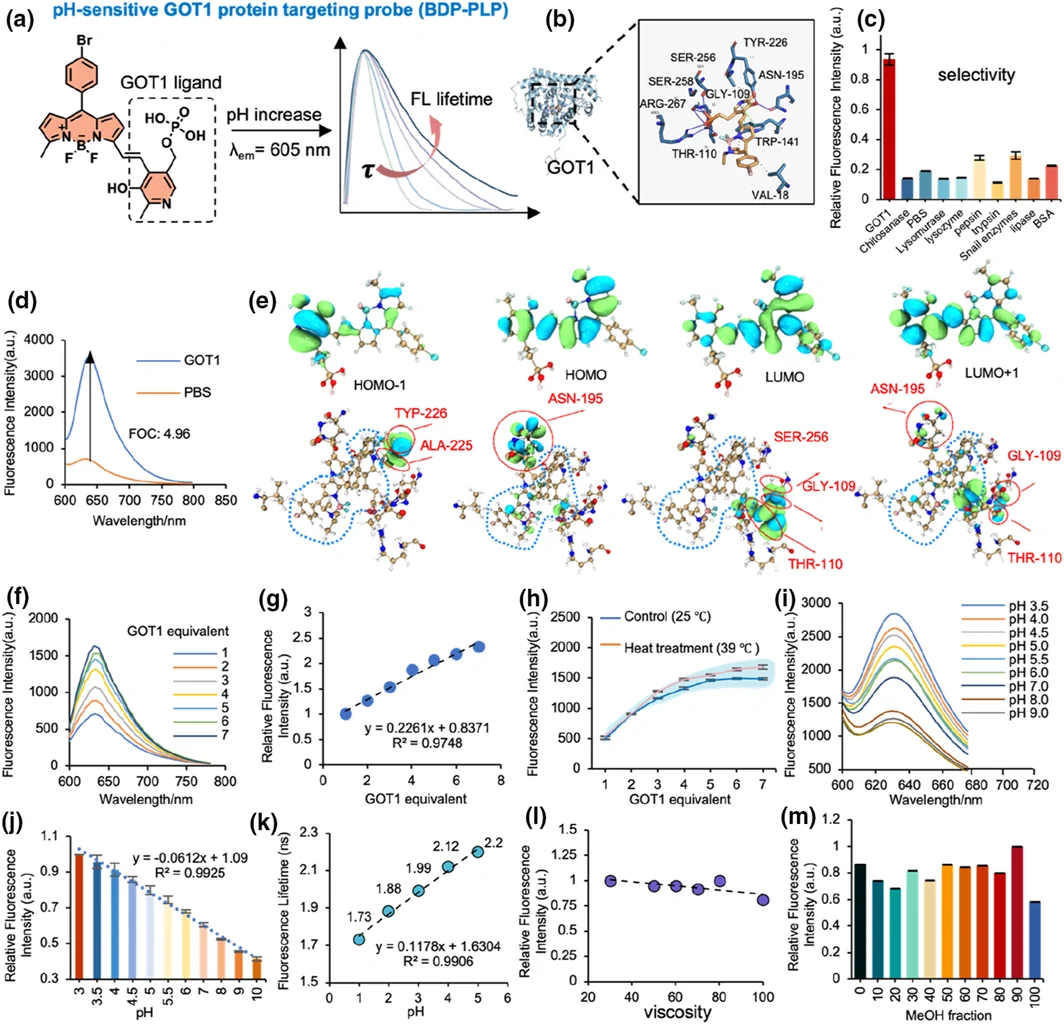
Design and characterization of BDP‐PLP, a GOT1‐Targeted, pH‐responsive fluorescence lifetime probe. (a) Chemical structure of the probe, formed by covalently linking the BODIPY fluorescent core to the native coenzyme pyridoxal phosphate of GOT1 via a Knoevenagel condensation reaction. (b) Molecular docking between the probe BDP‐PLP and human GOT1 protein, as well as a detailed view of key interactions at the binding interface. The PLP module forms a stable hydrogen‐bond network with multiple amino acid residues in the GOT1 active site, including GLY‐109, SER‐256, VAL‐18, ASN‐195, and TRP‐141. (c) Protein selectivity testing of the probe. Bar chart showing fluorescence intensity of the BDP‐PLP probe in PBS buffer (control) and after incubation with different proteins. (d) Fluorescence intensity changes of the probe before and after detecting GOT1. (e) Molecular orbital diagrams of probe BDP‐PLP before and after protein binding calculated by computational chemistry analysis. (f) Protein titration experiment. (g) Linear fitting calibration curve of the protein titration experiment. (h) Stability of the probe for protein detection at room temperature and physiological temperature. (i, j). In vitro pH‐responsive assay, fluorescence emission spectrum of BDP‐PLP in buffered solutions ranging from pH 3.0–10.0. (k) Calibration curve of the probe's fluorescence lifetime across different pH buffers. (l) Viscosity sensitivity assay of BDP‐PLP. (m) Polarity sensitivity assay of probe BDP‐PLP.

To decipher the interplay between intracellular pH fluctuations and GOT1‐mediated cellular senescence, and enable dynamic, quantitative imaging of pH in the local microenvironment of GOT1 within senescent cells, we engineered a dual‐functional fluorescent probe (BDP‐PLP) based on the boron dipyrromethene (BODIPY) fluorophore scaffold.[^44^, ^45^, ^46^] As illustrated in Figure 5a, to achieve fluorescence‐specific detection of GOT1, we incorporated pyridoxal phosphate (PLP), the endogenous coenzyme of GOT1,^[47]^ as both a specific targeting moiety and a pH‐responsive module. This was accomplished through Knoevenagel condensation with the BODIPY fluorophore core, endowing the probe with a high signal‐to‐noise ratio (SNR) response to pH changes at the target site and favorable photophysical properties (Supporting Information S1 Table S4).

Molecular docking simulations confirmed that BDP‐PLP binds specifically to the canonical PLP‐binding pocket of GOT1. In‐depth analysis of the binding mode revealed that the PLP module of the probe forms a stable hydrogen‐bonding network with critical residues in the active site, including GLY‐109, SER‐256, VAL‐18, ASN‐195, and TRP‐141 (Figure 5b). This provides a structural rationale for the probe's targeting specificity. To further validate the binding mode and stability of the BDP‐PLP–GOT, we performed a 100‐ns molecular dynamics (MD) simulation, followed by MM–PBSA binding free energy calculations. As shown in Supporting Information S1 Figure S15a, the root‐mean‐square deviation (RMSD) of the protein backbone reached equilibrium after approximately 20 ns and remained stable throughout the simulation, with an average RMSD of 0.422 ± 0.034 nm. Cα RMSF analysis (Supporting Information S1 Figure S15b) showed that most residues exhibited low flexibility (mean RMSF = 0.111 nm), indicating that GOT1 maintained a stable and compact conformation throughout the simulation. The average root‐mean‐square deviation (RMSD) of the ligand after protein fitting was 0.310 ± 0.047 nm (Supporting Information S1 Figure S15c), the average minimum distance between the protein and the ligand was 0.168 nm and remained below 0.2 nm throughout the simulation (Supporting Information S1 Figure S15d), and the average number of contacts was 196.96 (Supporting Information S1 Figure S15e). Hydrogen bond analysis showed that BDP‐PLP formed an average of 3.16 hydrogen bonds with GOT1, of which approximately 3 were maintained in the final frame (Supporting Information S1 Figure S15f). This indicates that BDP‐PLP was stably bound within the active site of GOT1 throughout the simulation. MM‐PBSA binding free energy calculations showed that the total binding free energy (ΔTOTAL) was −27.60 ± 6.91 kcal/mol (Supporting Information S1 Figure S15g), the gas‐phase interaction energy (ΔGGAS) was −120.62 kcal/mol, van der Waals interactions (ΔVDWAALS = −50.82 kcal/mol) and electrostatic interactions (ΔEEL = −69.80 kcal/mol) drove the binding, while the polar solvation energy (ΔEPB = +98.26 kcal/mol) counteracted this effect (Supporting Information S1 Figure S15h). An energy decomposition analysis by residue (Supporting Information S1 Figure S15i) identified the key residues ASP223, TRP141, PHE361, VAL18, TYR226, ALA225, THR110, VAL38, and SER258, which is consistent with the hydrogen‐bond network determined in the docking study. This demonstrates that BDP‐PLP binds to GOT1 in a specific and stable manner.

To verify the specificity of the probe's interaction with GOT1, we evaluated the probe's fluorescent response to GOT1 and other unrelated proteins. As shown in Figure 5c, a significant increase in fluorescence was observed only in the presence of wild‐type GOT1, whereas virtually no response was detected for the other proteins tested, confirming that the probe exhibits high target specificity.

Computational analysis revealed that the underlying photophysical mechanism of BDP‐PLP is based on a binding‐inhibited photoinduced electron transfer (PET) process. In an aqueous solution in the absence of GOT1, the probe exhibits weak fluorescence owing to an intramolecular PET process. Upon specific binding within the hydrophobic active site of GOT1, the probe engages in interactions with key amino acid residues. In particular, electron transfer occurs from the HOMO of the probe to Asn‐195, while electrons from the LUMO are transferred to Ser‐256, Gly‐109, and Thr‐110. The suppression of PET results in a marked enhancement of the fluorescence emission (Figure 5d,e, Supporting Information S1 Figure S10). This design ensures minimal background signal and a high signal‐to‐noise ratio for imaging GOT1 in living cells. Protein titration experiments demonstrated a linear increase in the fluorescence intensity of BDP‐PLP with increasing protein concentration (*R* ^2^ = 0.9748, Figure 5f,g). Furthermore, the experimental results indicate that the probe's limit of detection (LOD) is 0.9164 μM and its limit of quantification (LOQ) is 3.0547 μM. These demonstrate that BDP‐PLP possesses sufficient sensitivity to detect GOT1 at physiologically relevant concentrations (*R* ^2^ = 0.93; Supporting Information S1 Figure S16). Moreover, under physiological temperature conditions, the probe exhibited excellent stability in its sensing performance toward GOT1 (Figure 5h). These findings establish BDP‐PLP as a suitable molecular tool for visualizing GOT1 in live‐cell imaging applications.

To elucidate the pH‐responsive behavior of BDP‐PLP, we first characterized its fluorescence properties across a broad pH range (3.0–10.0) in buffer systems. The fluorescence intensity of BDP‐PLP exhibited a gradual pH‐dependent decrease with increasing pH (Figure 5i,j, Supporting Information S1 Figure S11). In parallel, fluorescence lifetime measurements revealed a linear positive correlation with pH (Figure 5k, Supporting Information S1 Figure S12), suggesting that the probe is capable of ratiometric or lifetime‐based pH sensing. We attribute this responsiveness to pH‐dependent modulation of radiative transition rates, governed by the protonation state of the molecule. Under acidic conditions, protonation of the ionizable groups extends the conjugation system of the BDP core, thereby enhancing fluorescence. In this case, the de‐excitation process of the excited‐state molecules back to the ground state is dominated by radiative transitions. Consequently, the radiative decay rate (*k*_r_) becomes predominant, leading to an accelerated total decay rate and thus a shortened fluorescence lifetime. In contrast, under alkaline conditions, the reduced conjugation weakens the fluorescence intensity, accompanied by a decrease in *k*_r_ and a slower total decay rate, which consequently prolongs the fluorescence lifetime (Figure 5k). To exclude the influence of the solvent microenvironment, we further evaluated the probe's response to variations in polarity and viscosity. No significant changes in fluorescence behavior were observed (Figure 5l,m, Supporting Information S1 Figure S13) and the fluorescence lifetime of the probe was also unaffected by the solvent microenvironment (Supporting Information S1 Figure S17), indicating that the pH‐dependent response arises primarily from protonation‐induced alterations in the conjugated system rather than from changes in environmental polarity or viscosity.

Finally, we validated the probe's functionality in live cells. BDP‐PLP was co‐incubated with GFP‐tagged GOT1 in cells, and confocal laser scanning microscopy (CLSM) imaging showed high signal colocalization between the two, with a Pearson correlation coefficient (PCC) of 0.84. This confirms the probe's specific targeting and labeling capability for GOT1 within the complex intracellular milieu (Supporting Information S1 Figure S14).

Collectively, we successfully developed and comprehensively characterized the BDP‐PLP probe. Its high targeting specificity, reversible pH responsiveness, and critical linear fluorescence lifetime‐pH relationship collectively validate BDP‐PLP as a robust chemical tool for investigating dynamic local pH fluctuations in the vicinity of GOT1 at subcellular resolution during physiological and pathological processes, including cellular senescence.

### 2.6 Fluorescence lifetime imaging‐enabled quantitative assessment of intracellular pH

Precise spatiotemporal regulation of intracellular pH is critical for sustaining diverse metabolic activities and fundamental cellular life processes. Accumulating evidence demonstrates that intracellular pH levels tend to decrease in senescent cells. Based on our previous findings that intracellular overexpression of the GOT1 protein mitigates growth defects induced by pH decline via inducing protein phase separation, the present study further developed a pH‐responsive fluorescence lifetime probe. This probe displays a decrease in fluorescence intensity with increasing pH, accompanied by a corresponding extension of its fluorescence lifetime, exhibiting excellent pH responsiveness.

Notably, the fluorescence lifetime is inherently independent of probe concentration, photobleaching, and excitation intensity, rendering it a robust quantitative imaging modality for reliable intracellular measurements. Leveraging this advantage, we employed fluorescence lifetime imaging microscopy (FLIM) to dynamically monitor intracellular pH and investigate its spatiotemporal dynamics in the context of cellular aging.

**FIGURE 6:**
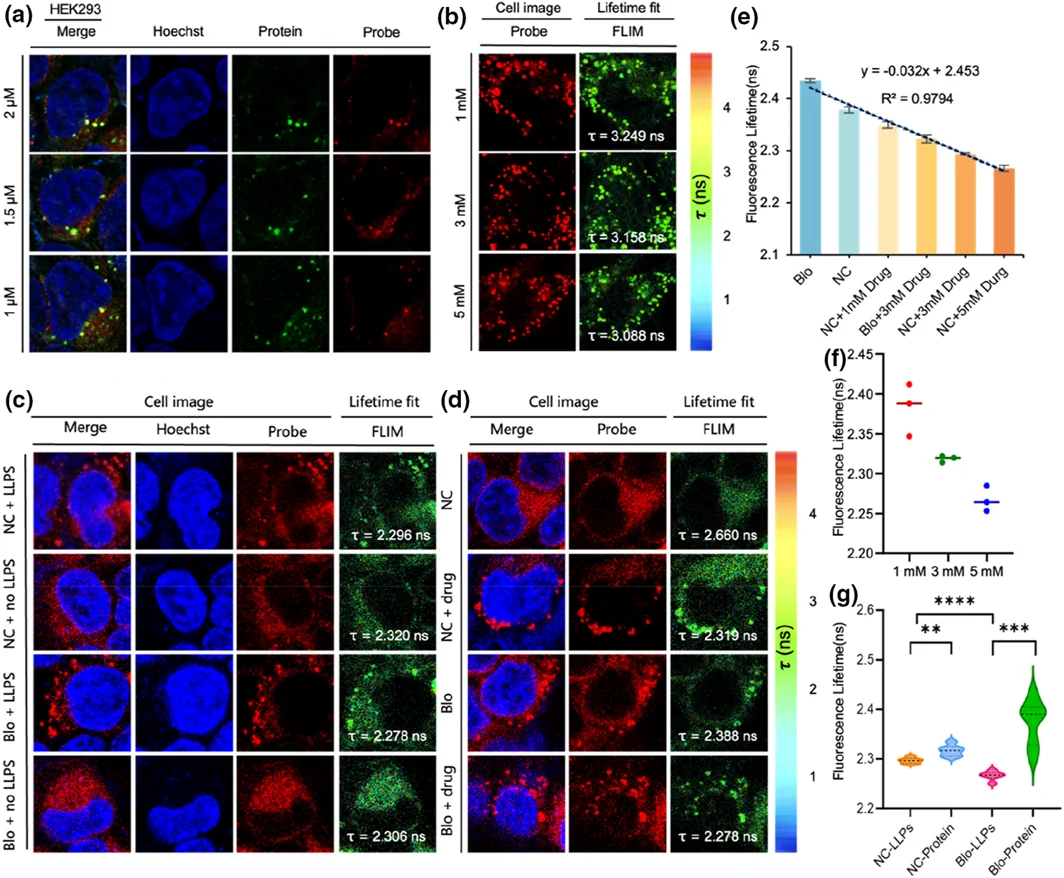
Quantitative pH mapping of senescence and GOT1 condensates via FLIM with BDP‐PLP. (a) Confocal image of probe‐labeled GOT1 droplets; scale bar: 10 μm. (b) Phase separation of intracellular proteins under stimulation by different drug concentrations. The fluorescence lifetime of the condensate varies with pH. (c) Quantitative histograms of the fluorescence lifetimes of dispersed proteins and protein condensates in young and aged cells following phase separation. The fluorescence lifetime in the undissociated regions of young cells (*τ* = 2.320 ns) was significantly higher than that in the phase‐separated droplets (*τ* = 2.296 ns; *p*‐value = 0.0031). In aged cells, the fluorescence lifetimes of both dispersed proteins and phase‐separated proteins exhibited significant differences (*p*‐value = 0.0002). (d) FLIM images (false color) of the probe quantitatively compared the average fluorescence lifetimes of young cells and bleomycin‐induced senescent cells, as well as FLIM imaging of intracellular pH in cells with and without protein phase separation. The average fluorescence lifetime of aged cells (*τ* = 2.388 ns) was significantly lower than that of young cells (*τ* = 2.660 ns; *p* < 0.0001, *t*‐test), confirming the overall acidification of the aged cytoplasm. Scale bar: 10 μm. The fluorescence lifetime of the condensate (*τ* = 2.278 ns) was shorter than the average lifetime of the whole cell (*τ* = 2.660 ns). (e) Fluorescence lifetime calibration curve converting the lifetimes measured by FLIM in (b–d) to absolute pH values. Bar charts provide a visual comparison of specific pH values across different regions (young cells, aged cells, inside GOT1 condensates, and surrounding cytoplasm). For FLIM imaging and fluorescence lifetime quantification, a calibration curve correlating fluorescence lifetime (τ) with absolute pH was established. (f) Quantitative analysis of the fluorescence lifetimes of droplets stimulated by different drug concentrations. (g) FLIM of the probe quantitatively compares the average fluorescence lifetime in young cells versus bleomycin‐induced senescent cells, as well as FLIM imaging of intracellular pH in cells with and without protein phase separation. The average fluorescence lifetime in aged cells (*τ* = 2.388 ns) was significantly lower than that in young cells (*τ* = 2.660 ns; *p* value < 0.0001, *t*‐test).

We first confirmed that the BDP‐PLP probe specifically labeled GOT1 and enabled visualization of GOT1‐enriched condensates formed via liquid–liquid phase separation (LLPS) in living cells (Figure 6a). Furthermore, cytotoxicity assays demonstrated that the BDP‐PLP probe exhibited very low cytotoxicity at concentrations effective for cell imaging (Supporting Information S1 Figure S18). To model the aged phenotype, cells were treated with bleomycin. FLIM analysis revealed a marked reduction in the average fluorescence lifetime of the probe in aged cells (*τ* = 2.388 ns, corresponding to ∼pH 6.4) compared to young cells (*τ* = 2.660 ns, *p* = 0.0031, ∼pH 7.0), indicating a global cytoplasmic acidification associated with senescence.

Notably, upon induction of GOT1 LLPS and subsequent formation of biomolecular condensates, a further decrease in fluorescence lifetime was observed within these microdomains (from *τ* = 2.319 ns to *τ* = 2.278 ns), suggesting that phase separation is accompanied by localized acidification (Figure 6d,g). These findings highlight the capacity of FLIM to resolve pH dynamics at subcellular resolution and reveal that both global and local pH shifts accompany aging and LLPS events.

To delineate the role of GOT1 phase separation in this acidification process, we overexpressed GOT1 in both young and aged cells and induced phase separation pharmacologically. As illustrated in Figure 6b, the fluorescence lifetime of the probe within the condensates (*τ* = 2.296 ns) was significantly lower than that in the surrounding diffuse regions (*τ* = 2.320 ± 0.050 ns; *p* = 0.0002). Notably, acidification within condensates formed in aged cells was more pronounced under identical induction conditions (Figure 6c,g, *p* = 0.0001).

Subsequently, analysis of GOT1 phase separation induced by gradient drug concentrations via FLIM imaging (Figure 6b) and quantitative assessments (Figure 6f) revealed that intracellular phase‐separated regions expanded with increasing drug concentration, accompanied by a concentration‐dependent decrease in overall fluorescence lifetime, with the lifetime of condensates decreasing from *τ* = 3.249 ± 0.117 ns to *τ* = 3.088 ± 0.106 ns. To convert fluorescence lifetime signals into absolute pH values, we established a fluorescence lifetime‐pH calibration curve through intracellular fluorescence lifetime calibration (Figure 6e). Using this calibration curve, we successfully quantified pH levels in young cells, senescent cells, and phase‐separated microdomains.

Collectively, by developing FLIM‐based quantitative pH imaging technology, this study delineates the spatiotemporal dynamics of intracellular pH during senescence and associated protein phase separation at the single‐cell level. This finding provides critical experimental evidence for deciphering the link between senescence‐associated metabolic dysregulation and the functional regulation of biomolecular condensates. Generally, acid stress induces GOT1 to recruit ME1, leading to the formation of dynamic metabolic condensates. This condensate acts as a functional unit to efficiently regenerate NADPH and scavenge intracellular ROS, thereby breaking the vicious cycle of oxidative stress associated with senescence and conferring a cytoprotective effect.

## 3 CONCLUSION

In conclusion, this study elucidates a cellular anti‐aging mechanism mediated by biomolecular phase separation in response to microenvironmental acidification. We demonstrate that the metabolic enzyme GOT1 undergoes liquid‐liquid phase separation via pH sensing through its intrinsically disordered N‐terminal region, which promotes co‐condensation with malate decarboxylase ME1. The resulting dynamic condensates function as reactive oxygen species scavengers, thereby attenuating cellular senescence. By employing a pH‐responsive fluorescence lifetime probe (BDP‐PLP), tailored for GOT1, we achieved specific protein labeling, and through fluorescence lifetime imaging microscopy (FLIM), quantitatively mapped local pH changes within GOT1 condensates in live cells based on an in vitro‐calibrated lifetime‐pH correlation. Our findings establish the essential role of pH‐driven GOT1 phase separation in regulating redox homeostasis and delaying aging, offering novel therapeutic targets and a conceptual framework for intervening in age‐related pathologies. Additionally, the molecular probes and imaging methodologies developed herein provide robust tools for further exploration of the physicochemical underpinnings of biological processes.

## AUTHOR CONTRIBUTIONS

Ziyue Chen was responsible for all experimental design and execution, data analysis, and manuscript writing. Jiaqi Li was responsible for fluorescence lifetime imaging. Yan Bao was responsible for data analysis. Mengmeng Wang was responsible for computational chemistry calculations. Haoyan Chen and Mingjiong Zhang were responsible for bioinformatics data analysis. Lina Ding was responsible for the method design and guidance of computational chemistry. Shuangshuang Wu was responsible for project guidance, manuscript writing and revision. Baoxing Shen was responsible for project design, experimental guidance, data analysis, manuscript writing and revision, and funding acquisition.

## CONFLICT OF INTEREST STATEMENT

The authors declare no conflicts of interest.

## ETHICS STATEMENT

Ethical approval for humans was granted by the Ethics Committee of the First Affiliated Hospital of Nanjing Medical University (ID: 2022‐SR‐177) for this study.

## Supporting information

Supporting Information S1

Table S1

Table S2

Table S3

## Data Availability

The data that support the findings of this study are available from the corresponding author upon reasonable request.
