# Supplementary material for: A pH‐Responsive fluorescence lifetime probe reveals GOT1 phase separation in cellular anti‐senescence
Source: Smart Mol. 2026 Sep 3:e70096. Online ahead of print. doi: 10.1002/smo2.70096 (PMC13539394; doi:10.1002/smo2.70096)
Supplement: Supplementary file 1 [file SMO2-9999-0-s004.docx]

**Supplementary information**

A pH-Responsive Fluorescence Lifetime Probe Reveals GOT1 Phase Separation in Cellular Anti-Senescence

Ziyue Chen,^[a][b]†^ Jiaqi Li,^[a][b]†^ Yan Bao,^[a][b]†^ Mengmeng Wang,^[d]^ Haoyan Chen, ^[c]^ Mingjiong Zhang, ^[c]^ Lina Ding, * ^[d]^ Shuangshuang Wu, * ^[c]^ and Baoxing Shen *^[a][b]^

Ziyue Chen, Jiaqi Li, Yan Bao, Baoxing Shen

[a] State Key Laboratory of Microbial Technology, Nanjing Normal University, Nanjing 210023, China

E-mail: shenbx@njnu.edu.cn

Ziyue Chen, Jiaqi Li, Yan Bao, Baoxing Shen

[b] School of Food Science and Pharmaceutical Engineering, Nanjing Normal University, Nanjing 210023, China

E-mail: shenbx@njnu.edu.cn

Haoyan Chen, Mingjiong Zhang, Shuangshuang Wu

[c] Jiangsu Provincial Key Laboratory of Geriatrics, Department of Geriatrics, The First Affiliated Hospital of Nanjing Medical University, Nanjing 210029, China.

E-mail: polariswu7632@njmu.edu.cn

Mengmeng Wang, Lina Ding

[d] School of Pharmaceutical Sciences, Zhengzhou University, Zhengzhou 450001, China
E-mail: [dinglina123@126.com](mailto:dinglina123@126.com)

* corresponding authors: Lina Ding (dinglina123@126.com), Shuangshuang Wu (polariswu7632@njmu.edu.cn), Baoxing Shen (shenbx@njnu.edu.cn)

†These authors contributed equally to this work.

**Table of Contents**

**1. Experimental Procedures**

1.1. Sample Collection and proteomics Analysis

1.2. Cell Culture

1.3. GOT1 Protein Disordered Region Prediction

1.4. Glutamic-oxaloacetic Transaminase Activity Test

1.5. In vitro phase-separation assay

1.6. FRAP analysis

1.7. β-Galactosidase staining

1.8. qRT-PCR analysis

1.9. Western blot analysis

1.10. Flow cytometry

1.11. Protein Expression and Purification

1.12. Measurement of fluorescence intensity

1.13. Plasmid transfection and confocal imaging

1.14. Lifetime Imaging

1.15. Molecular Dynamics Simulation

1.16. Determination of the Limit of Detection (LOD) for BDP-PLP Probes

1.17. Cell Viability Assay (CCK-8)

1.18. Photophysical Property Measurements

**2. Supplementary Tables**

2.1. Differential Gene Transcriptome Expression Analysis in Peripheral Blood Cells.

2.2. Differential Gene Expression Analysis in Serum Transcriptomes.

2.3. Raw Data for Differential Analysis of Protein Expression in Serum Samples from Young and Elderly Individuals.

2.4. The photophysical properties of BDP-PLP.

2.5. qPCR primers.

**3. Supplementary Figures**

**4. Synthetic Methods and Scheme**

**5. NMR Spectra of the Compounds**

**6. High resolution mass spectrometry (HRMS) of the compounds**

**1. Experimental Procedures**

**1.1 Sample Collection and proteomics Analysis**

In collaboration with Jiangsu Provincial People’s Hospital, serum and peripheral blood cell samples were collected from young and elderly individuals. Samples were grouped into early-stage or late-stage categories (n=3 per group; ***Ethical approval for human was granted by the Ethics Committee of the First Affiliated Hospital of Nanjing Medical University ID: 2022-SR-177 for this study***), processed, and subjected to subsequent proteomics analysis. Liquid chromatography-mass spectrometry (LC-MS) and raw data were provided by Lianchuang Biotechnology (Hangzhou, China), utilizing the nanoUPLC system EASYnLC1200 coupled with the Q Exactive HFX mass spectrometer (Thermo Fisher Scientific). Differential protein expression analysis was combined with the public aging database (https://geneyun.net/ADEIP/) to identify intersections. Volcano plots, heatmaps, and protein correlation analyses were performed using corresponding R software packages. Significantly enriched events were defined as differentially expressed with P-values ≤ 0.05.

**1.2 Cell culture**

BEAS-2B cells were routinely cultured in Dulbecco's Modified Eagle Medium (DMEM) supplemented with 10% fetal bovine serum (FBS) and maintained at 37°C in a humidified incubator with 5% CO₂. The culture medium was refreshed every 2–3 days, and cells were subcultured at 80–90% confluency by dissociation with trypsin-EDTA, followed by reseeding at a split ratio of 1:2 to 1:3. The cell line identity was confirmed by STR profiling, matching the reference database. For long-term storage, cells were cryopreserved in freezing medium consisting of 50% DMEM, 40% FBS, and 10% DMSO at a density of 1×10⁶ to 1×10⁷ cells/mL.

HEK-293 cells were routinely cultured in Dulbecco's Modified Eagle Medium (DMEM) supplemented with 10% fetal bovine serum (FBS) and maintained at 37°C in a humidified incubator with 5% CO₂. The cells, which exhibit an epithelial-like and adherent morphology, were subcultured at high confluency using 0.25% trypsin for digestion (2-3 minutes) and typically passaged at a 1:4 split ratio. For long-term preservation, cells were cryopreserved in a freezing medium consisting of 92% complete culture medium and 8% DMSO.

**1.3 GOT1 protein disordered region prediction**

The disordered region of the GOT1 protein was predicted using PONDR software analysis. (https://www.pondr.com/pondr-tut.html)

**1.4 Glutamic-oxaloacetic transaminase activity test**

GOT/AST activity was determined using a commercial assay kit (BC1565, Solaibao) according to the manufacturer's protocol. The assay is based on the enzymatic conversion of aspartate and α-ketoglutarate to oxaloacetate (and subsequently to pyruvate) by GOT. The generated pyruvate reacts with 2,4-dinitrophenylhydrazine to form a brown-red 2,4-dinitrophenylhydrazone derivative under alkaline conditions, the absorbance of which is measured at 505 nm. Briefly, harvested cells were lysed in the provided extraction buffer by sonication on ice, and the supernatant was collected after centrifugation (3,500 × g, 10 min, 4°C). In the assay, 5 µL of supernatant was mixed sequentially with Reagent 1 and Reagent 2, incubated at 37°C for 30 min and 20 min, respectively, followed by the addition of Reagent 3. After a 10-minute incubation at room temperature, absorbance was measured at 505 nm using a microplate reader. A standard curve was generated using pyruvate standards (0–1 µmol/mL) processed in parallel. GOT activity was calculated based on the amount of pyruvate produced per hour and normalized to cell number (U/10⁶ cells). All samples were assayed in duplicate, with appropriate sample and reagent blank controls included.

**1.5 In vitro phase-separation assay**

The recombinant protein GOT1-EGFP-his was first cleared of aggregates by ultracentrifugation (12,000 g, 4°C, 15 min). The protein was diluted into physiological buffer (50-150 mM NaCl, 20 mM Tris-HCl pH 7.4) and mixed with varying concentrations of molecular crowding agents (e.g., 10% PEG-8000) in a 1.5 ml centrifuge tube. After brief incubation (30 min), 5 μl of the mixture was dispensed onto a microscope slide. Observe droplets under fluorescence microscopy. Quantify droplet size, density, and fluorescence intensity via image analysis (ImageJ). To ensure reliability, experiments utilized freshly purified proteins and corresponding control groups.

**1.6 FRAP analysis**

For FRAP experiments, human embryonic kidney cells (HEK293) and human normal lung epithelial cells (BEAS-2B) infected with the GOT1-EGFP plasmid for 24–36 hours were seeded onto glass-bottom dishes and subsequently imaged using confocal microscopy. Two control images were acquired prior to bleaching. A selected fluorescent spot was bleached once at 60% laser transmission (488 nm laser), and droplet fluorescence recovery images were recorded post-bleaching. Under these conditions, approximately 70–80% of the signal was bleached. Time-lapse images were acquired every 15 seconds, and fluorescence intensity at the spot was measured using Nikon software.

**1.7 β-Galactosidase staining**

β-galactosidase staining for senescent cells was performed using a commercial kit, based on the biological property of senescent cells overexpressing β-galactosidase at pH 6.0. This enzyme catalyzes the hydrolysis of the substrate X-Gal in an acidic environment, producing an insoluble deep blue precipitate, thereby enabling the identification of senescent cells under optical microscopy. Brief procedure: After washing adherent cells with PBS, fix them at room temperature for 15 minutes using β-galactosidase staining fixative; Wash three times with PBS, each for 3 minutes; then add 1 mL of freshly prepared staining working solution to each well (composition: β-galactosidase staining solution A 10 μL, staining solution B 10 μL, staining solution C 930 μL, X-Gal solution 50 μL); Seal the culture plate and incubate overnight at 37°C in a CO₂-free incubator protected from light. The following day, observe and count blue-stained positive cells under an optical microscope. Throughout the staining process, avoid crystallization of the staining solution and exposure to light. Do not place the plate in a CO₂ incubator during incubation to prevent pH changes that could affect staining efficiency. This assay exhibits high specificity, effectively distinguishing senescent cells from quiescent or immortal cells.

**1.8 qRT-PCR analysis**

Total RNA was extracted from cells or tissues using the TRIzol reagent method. After determining concentration and purity via Nanodrop, 1 μg of RNA was used for reverse transcription into cDNA with the Novazene RT Kit. qRT-PCR was performed using the SYBR Green method on a QuantStudio system. The reaction mixture contained SYBR Premix Ex Taq II, specific primers, and cDNA template. Cycling conditions were as follows: 30 seconds at 95°C for pre-denaturation, followed by 40 cycles of 5 seconds at 95°C and 30 seconds at 60°C. A melting curve analysis was conducted at the end. All samples were processed in triplicate with a no-template control. Primers were professionally designed and synthesized, with GAPDH serving as the internal control gene. Relative gene expression was calculated using the 2^(−ΔΔCt) method. Data are presented as mean ± standard deviation and analyzed statistically using GraphPad Prism software.

**1.9 Western blot analysis**

Protein samples were extracted from BEAS-2B cells using lysis buffer (containing RIPA, PMSF, and protease inhibitors) on ice. Protein concentration was determined by the BCA method. Equal amounts of protein (typically 20–30 μg) were mixed with 6× loading buffer and denatured by heating at 100°C for 5 minutes. Proteins were separated by electrophoresis on a 15% SDS-polyacrylamide gel. Initial voltage was set at 80 V, then increased to 120 V once samples entered the separating gel. After electrophoresis, proteins were transferred to a PVDF membrane via wet transfer at a constant current of 200 mA for 90 minutes. After transfer, the membrane was blocked with 5% nonfat milk at room temperature for 1 hour. It was then incubated overnight at 4°C with primary antibodies (GAPDH and p21 antibodies diluted according to Abcam protocols). Following three washes with TBST (10 min each), the membrane was incubated at room temperature for 1.5 hours with species-specific horseradish peroxidase-labeled secondary antibodies. After another wash, uniformly coat the membrane with ECL chemiluminescent substrate and capture images using a chemiluminescence imaging system. All images undergo grayscale analysis using ImageJ software. Target protein expression levels are normalized against the reference protein and subjected to statistical comparison.

**1.10 Flow cytometry**

Intracellular ROS levels were detected using the fluorescent probe DCFH-DA. BEAS-2B cells were seeded at appropriate density in six-well plates and cultured to approximately 70% confluence. Following treatment under different conditions, the medium was removed, and cells were gently washed twice with pre-warmed PBS. Each well was then added 1 mL of freshly prepared 10 mM DCFH-DA working solution in serum-free medium and incubated at 37°C in a cell culture incubator, protected from light, for 30 minutes. After incubation, cells were washed twice with pre-warmed PBS to thoroughly remove unincorporated probe. Cells were then digested with 0.25% trypsin (EDTA-free) and collected into flow cytometry tubes. Cells were resuspended in pre-chilled PBS and placed on ice. Detect the mean fluorescence intensity (MFI) of at least 10,000 cells using flow cytometer at an excitation wavelength of 488 nm and an emission wavelength of 525 nm. The positive control well was treated with an appropriate concentration of hydrogen peroxide for 1 hour prior to probe incubation. All experiments were independently replicated three times. Results were expressed as fold changes in fluorescence intensity relative to the control group and statistically analyzed using FLOW JO software.

**1.11 Protein expression and purification**

Expression and Purification of Human GOT1 Protein. The GOT1 gene sequence was cloned into the pET-22b(+) expression vector to construct the recombinant plasmid pET22b-got1 bearing a 6×His tag at the C-terminus. This plasmid was transformed into E. coli BL21(DE3) strain. Cultures were grown in LB medium containing 100 μg/mL ampicillin at 37°C until an OD₆₀₀ of 0.6 was reached. 1 mM IPTG was then added, and expression was induced at 30°C for 6 hours. The cell pellet was collected by centrifugation, resuspended in Buffer A (20 mM NaH₂PO₄, 0.5 M NaCl, pH 7.4), and sonicated on ice. The lysate was centrifuged at 4°C and 12,000 rpm for 15 minutes. The supernatant was filtered through a 0.45 μm membrane and loaded onto a pre-equilibrated HisTrap FF crude nickel column. The column was first washed with Buffer A containing 20 mM imidazole, followed by elution of the target protein with Buffer B (20 mM NaH₂PO₄, 0.5 M NaCl, 250 mM imidazole, pH 7.4) to elute the target protein. The eluate was exchanged into 20 mM Tris-HCl (pH 7.0, containing 20 mM NaCl) buffer via a NiTrap desalting column, then loaded onto a Ni column and collected by elution with a 0–500 mM imidazole linear gradient. The protein was desalted again into storage buffer (20 mM HEPES, pH 7.5, 20 mM NaCl) and concentrated using a 30 kDa ultrafiltration centrifuge tube. Protein purity was assessed by SDS-PAGE (>95%) and is suitable for subsequent biochemical analysis.

**1.12 Measurement of fluorescence intensity**

For the BODIPY specificity analysis, each test system contained 10μM of specified probe and 46μM GOT1 protein in 200μL buffer solution (20mM NaH₂PO₄, pH 7.5, 100 mM NaCl). For the protein specificity analysis, each test system contained 2 μM of BODIPY and 50 μM specified protein in 200 μL buffer solution (20mM NaH₂PO₄, pH 7.5, 100 mM NaCl). For the protein titration analysis, each test system contained 2μM of BODIPY and 0-8 μM GOT1 protein in 200μL buffer solution (50 mM Tris-HCl, pH 7.5, 100 mM NaCl). For the polarity pH sensitivity analysis, 2μM of BODIPY was added to solvents with different pH mixtures of specified ratios. Each mixture was mixed thoroughly before measuring its peak fluorescence intensity.

**1.13 Plasmid transfection and confocal imaging**

Cell transfection requires seeding HEK293 and BEAS-2B cells in logarithmic growth phase at appropriate densities (HEK293: 5–8 × 10⁵ cells/well; BEAS-2B: 3–5 × 10⁵ cells/well) at appropriate densities into 6-well plates using complete medium. Incubate overnight at 37°C in a 5% CO₂ incubator to achieve 70–90% confluence at the time of transfection. For transfection, prepare a DNA-liposome complex for each well containing cells: Dilute 1–2 μg of plasmid DNA in 50 μL Opti-MEM® serum-free medium and gently mix. In a separate tube, dilute 2 μL Lipofectamine™ 3000 reagent in 50 μL Opti-MEM® and incubate at room temperature for 5 minutes. Promptly mix the diluted plasmid DNA solution with the diluted Lipofectamine™ 3000 reagent and incubate at room temperature for 15 minutes to form the transfection complex. After complex formation, add the complex dropwise to the corresponding cell culture wells and gently shake the plate to mix. Incubate cells with 1.5 mL fresh antibiotic-free complete medium. Return cells to the incubator for continued culture. Fresh complete medium (with or without antibiotics) may be added 6 hours post-transfection. Transfection efficiency can be assessed by fluorescence microscopy 24-48 hours post-transfection. For BEAS-2B cells, due to their relative sensitivity to transfection stress, the transfection reagent volume should be reduced to 1 μL, and cell status should be closely monitored post-transfection.

Imaging was carried out with Nikon AX confocal microscope.

**1.14. Lifetime imaging**

Refer to the previous section for cell culturing and plasmid transfection. Fluorescent lifetime signals are recorded by VistaVision FastFLIM.

**1.15 Molecular Dynamics Simulation**

Molecular dynamics simulations of the protein-ligand complex were performed using GROMACS 2024.3. The protein topology was generated using `gmx pdb2gmx`; the Amber99sb-ildn force field and the TIP3P water model were used. The ligand topology was generated using ACPYPE and then merged into the GROMACS topology file. The system was placed in a periodic cubic water box, and 0.15 M Na⁺ was added to electrically neutralize the system. The simulation system was first energy-minimized using the steepest descent algorithm for a maximum of 5,000 steps. This was followed by a 100 ps equilibrium simulation with a time step of 1 fs, during which positional constraints were applied to the system. During the equilibration phase, temperature coupling was performed using the V-rescale method with a reference temperature of 298.15 K, and pressure coupling was performed using the Berendsen method with a reference pressure of 1.0 bar. The main simulation employed the NPT ensemble with an integration step size of 2 fs and a total of 50,000,000 steps, corresponding to a simulation time of 100 ns. For the production phase, temperature coupling was performed using the V-rescale method, and pressure coupling was performed using the Parrinello-Rahman method with isotropic pressure coupling and a reference pressure of 1.0 bar. Long-range electrostatic interactions were modeled using the PME method, with both the Coulomb and van der Waals cutoff radii set to 1.0 nm, and periodic boundary conditions were applied. Constraints related to hydrogen bonds were set to hbonds.

After the simulation, the trajectories were subjected to periodic boundary condition correction, complex centering, and protein-based structure fitting. Hydrogen bond analysis was performed using gmx hbond-legacy. The binding free energy was calculated using gmx_MMPBSA version 1.6.4. The calculation used the PB model, with a salt concentration of 0.15 M, fillratio=4.0, and radiopt=0.

**1.16 Determination of the Limit of Detection (LOD) for BDP-PLP Probes**

The limit of detection (LOD) and limit of quantification (LOQ) for GOT1 using this probe were determined through fluorescence titration experiments. Purified GOT1 protein was serially diluted in PBS buffer (10 mM, pH 7.4) to yield final concentrations of 0 (blank), 0.156, 0.3125, 0.675, 1.25, 2.5, 5, 10, and 20µM. Add the BDP-PLP probe to each GOT1 solution to achieve a fixed final concentration of 2 µM. Incubate the mixture at room temperature in the dark for 40 minutes to ensure complete binding, then record the fluorescence emission spectrum using a fluorometer at 25 °C with an excitation wavelength of 500 nm (slit width: 5 nm; PMT voltage: 800 V). Three replicates were performed for each concentration (n = 3). The net fluorescence intensity (ΔF = F sample – F blank) was plotted against GOT1 concentration, and a linear regression analysis was performed within the linear range of the calibration curve. The standard deviation (σ) of the blank sample (PBS buffer containing 2 µM BDP-PLP but no GOT1) was determined through 10 replicate measurements. The limit of detection (LOD) and limit of quantification (LOQ) were calculated as LOD = 3σ/k and LOQ = 10σ/k, respectively, where k is the slope of the linear calibration curve. All measurements were performed under light-shielded conditions.

**1.17 Cell Viability Assay (CCK-8)**

Use the Cell Counting Kit-8 to assess the cytotoxicity of the BDP-PLP probe in the HEK293 cell line. Harvest cells in the logarithmic growth phase and seed them into a 96-well plate at a density of 5 × 10³ cells per well using 100 µL of complete medium. Incubate the culture plate for 24 hours to allow the cells to attach and reach approximately 70–80% confluence. Next, replace the culture medium with fresh medium containing different concentrations of BDP-PLP (0, 0.5, 1, 1.5, 3, 4, 6, and 10 µM). Add an equal volume of dimethyl sulfoxide (DMSO, final concentration ≤ 0.1%) to the control wells as a control. Test each concentration in three replicate wells. The cells were then cultured for 1 hour at 37 °C in a 5% CO₂ atmosphere. The culture medium was aspirated, the cells were washed with PBS, and fresh culture medium was added. Next, 10 µL of CCK-8 solution was added, and the plate was incubated at 37 °C for 1 hour. Absorbance at 450 nm was measured using a microplate reader.

Cell survival rate (%) = [(A₄₅₀(sample) – A₄₅₀(blank)) / (A₄₅₀(control) – A₄₅₀(blank))] × 100% (where A₄₅₀(sample) and A₄₅₀(control) represent the absorbance values of cells treated with BDP-PLP and solvent, respectively). )

All data represent the mean ± standard deviation (SD) from three independent experiments. Statistical analysis was performed using one-way analysis of variance (ANOVA), followed by post-hoc tests using GraphPad Prism 9.0 software. A cell survival rate above 90% was considered to indicate no significant cytotoxicity.

**1.18. Photophysical Property Measurements**

UV-visible absorption spectra were measured at 25 °C using a UV-visible spectrophotometer with a 1-cm-pathlength cuvette. BDP-PLP was dissolved in ethanol to prepare a 5 μM solution. Absorption spectra were recorded in the wavelength range of 400–700 nm. According to the Beer–Lambert law (A = ε·c·l), the molar extinction coefficient (ε) was calculated from the slope of the linear regression between absorbance and concentration.

Fluorescence emission spectra were measured at 25 °C using a fluorescence spectrophotometer (Hitachi F-7100) and a 1-cm pathlength quartz cuvette. The excitation wavelength was set to the absorption peak, and emission spectra were recorded in the 500–700 nm wavelength range. Both the excitation and emission slit widths were 5 nm, and the photomultiplier tube (PMT) voltage was 800 V. The Stokes shift was calculated as the difference between the emission peak and the absorption peak: Δf = λ_em_ – λ_abs_.

The fluorescence quantum yield (Φ) was determined using the relative method, with Rhodamine B (Φ_std = 0.65, ethanol solution, calibrated using a water:ethanol mixture in a 4:1 ratio) serving as the reference standard. A series of sample and standard solutions were prepared, and the quantum yields were calculated using the following equation:

φF(X): Probe fluorescence quantum yield; φF(S): Standard fluorescence quantum yield; A: Absorbance at the excitation wavelength; F: Area under the emission curve; X: Probe; S: Standard; Ƞ: Solvent refractive index

Luminance is the product of the molar extinction coefficient (ε) and the fluorescence quantum yield (Φ): Luminance = ε × Φ.
**2. Supplementary Tables**

**Supplementary Table S1**

Differential Gene Transcriptome Expression Analysis in Peripheral Blood Cells

**Supplementary Table S2**

Differential Gene Expression Analysis in Serum Transcriptomes

**Supplementary Table S3**

Raw Data for Differential Analysis of Protein Expression in Serum Samples from Young and Elderly Individuals

**Supplementary Table S4**

The photophysical properties of BDP-PLP

| Compounds | λ_em_^a^ (nm) | Φ^b^ | λ_abs_^c^ (nm) | ε^d^ (M^−1^cm^−1^) | B^e^ (M^−1^cm^−1^) | Δλ^f^ (nm) |
| --- | --- | --- | --- | --- | --- | --- |
| BDP-PLP | 625 | 0.6101 | 604 | 1.66 × 10⁴ | 1.013× 10⁴ | 21 |

^a^ Emission wavelength (nm).

^b^ Fluorescence quantum yield.

^c^ Absorbance wavelength (nm).

^d^ Molar extinction coefficient (M^−1^cm^−1^).

^e^ Brightness (B) (M^−1^cm^−1^).

^f^ Stokes shift (nm).

Fluorescence quantum yields of BDP-PLP under different conditions

| Conditions | pH=5 | pH=6 | pH=7 | pH=8 |
| --- | --- | --- | --- | --- |
| Φ | 0.66 | 0.632 | 0.6101 | 0.42 |

**Supplementary Table S5**

qPCR primers

| Name | Forward primer | Reverse primer |
| --- | --- | --- |
| P21 | GCCGAAGTCAGTTCCTTGTG | TTCTGACATGGCGCCTCCT |
| GOT1 | TCGTGCGGATTACTTGGTCC | CTCAACCTGCTTGGGGTTCA |
| GAPDH | GTCTCCTCTGACTTCAACAGCG | ACCACCCTGTTGCTGTAGCCAA |

**3. Supplementary Figures**

**3.1 Proteomics Analysis of Peripheral Blood Cells and serum**

**Figure S1.** GOT1 expression levels in serum samples from young and elderly subjects.

**Figure S2****.** Volcano plot showing differences in GOT1 expression in peripheral blood cells, (p-value < 0.05, fold change > 1.5).

**Figure S3** Expression levels of GOT1 in peripheral blood cells from young and elderly samples.

**3.2 SDS-PAGE**

**Figure S4.** SDS-PAGE gel image of purified GOT1. Lane 1 is the protein marker. The molecular weight of GOT1 is 42 kDa.

**3.3 Phase separation of GOT1 in cells and *in vitro***

**Figure S5.** Phase separation behavior of GOT1 in PEG1000 solutions at 10%, 30%, and 50% concentrations.

**Figure S6.** The phase separation of intracellular GOT1 under stimulation by different drug concentrations.

**3.4 Intracellular Fluorescence Recovery After Photobleaching (FRAP)**

**Figure S7.** Protein photobleaching recovery data, with a recovery rate of 67.8%.

**3.5 Western Blots**

**Figure S8.** Verify p21 expression levels within cells following GOT1 phase separation using Western blot analysis. Control represents untreated cells. Lower indicates cells with suppressed GOT1 expression. Lower+drug denotes cells treated with drugs after GOT1 suppression. Overexpress signifies cells with elevated GOT1 expression. Overexpress+drug represents cells overexpressing GOT1 where phase separation has occurred.

**Figure S9.** Western blot validation of the effect of knocking out the two disordered regions in GOT1 on intracellular P21 expression. WT-GOT1 represents wild-type GOT1, GOT1-ΔIDR1 represents GOT1 with the first disordered region knocked out, and GOT1-ΔIDR2 represents GOT1 with the second disordered region knocked out.

**3.6** **Spectral diagram of probe BDP-PLP in GOT1 protein solution and PBS (pH=7.4)**

**Figure S10.** Fluorescence spectra of the probe in GOT1 protein solution (pH=7.4) and PBS (pH=7.4).

**3.7 pH Response Spectrum of Probe BDP-PLP**

**Figure S11.** Fluorescence spectra of probe BDP-PLP in solutions of different pH values.

**3.7 Fluorescence Lifetime Testing of Probes**

**Figure S12.** Fluorescence lifetime of probe BDP-PLP at different pH levels.

**3.9 Polarity Testing of Probes in different Solvents**

**Figure S13.** Fluorescence intensity testing of BDP-PLP in different solutions.

**3.10 Intracellular confocal imaging of cells labeled with the GOT1 probe**

**Figure S14.** Intracellular imaging of GOT1 labeled with probes of varying concentrations and Pearson correlation coefficient analysis

**3.11 Molecular Dynamics Simulations and MM/PBSA Analysis Results**

**Figure S15.** Molecular dynamics simulations of the BDP-PLP–GOT1 complex and MM-PBSA binding free energy analysis. (a) Root-mean-square deviation (RMSD) of the protein main chain. (b) Root-mean-square fluctuation (RMSF) of the Cα of each residue. (c) Root-mean-square deviation (RMSD) of the ligand after protein fitting. (d) Minimum distance between the protein and the ligand. (e) Number of intermolecular contacts between the protein and the ligand. (f) Number of hydrogen bonds formed between BDP-PLP and GOT1. (g) MM-PBSA binding free energy components. (h) Energy decomposition by interaction type. (i) Energy decomposition analysis by residue, showing the contribution of key residues within 4 Å of the ligand to the binding free energy. Simulations were performed using GROMACS 2024.3 software with the amber99sb-ildn force field and the TIP3P water model. MM-PBSA calculations were performed using gmx_MMPBSA 1.6.4 software, with 101 frames extracted at 1 ns intervals from a 100 ns trajectory. Data are presented as mean ± standard deviation (SD).

**3.12 Molecular Dynamics Simulations and MM/PBSA Analysis Results**

**Figure S16.** Determination of the limit of detection (LOD) and limit of quantification (LOQ); standard calibration curve showing the linear relationship between net fluorescence intensity and GOT1 concentration. Data are presented as mean ± SD (n = 3). The LOD was calculated using the formula LOD = 3σ/k, and the LOQ was calculated using the formula LOQ = 10σ/k, where σ is the standard deviation of 10 replicate measurements of blank samples.

**3.13 Fluorescence Lifetime Measurements of the BDP-PLP Probe under Different Viscosity and Polarity Conditions**

**Figure S17.** Fluorescence lifetime measurements of the probe. (a–b) Fluorescence lifetime of BDP-PLP under different polarity conditions; (c–d) Changes in the fluorescence lifetime of BDP-PLP under different viscosity conditions.

**3.14 Cell Counting Kit-8 (CCK-8) experiment**

**Figure S18**. Assessment of the cytotoxicity of the BDP-PLP probe in HEK293 cells. Cells were treated with gradually increasing concentrations of BDP-PLP (0–10 μM) for 1 hour, and cell viability was assessed using the CCK-8 assay. Data are presented as mean ± standard deviation (n = 3).

**3.15 Bioinformatics analysis**

**Figure S19.** Bioinformatics analysis of the physicochemical properties of GOT1, IDR1, and IDR2. (a) Charge composition analysis of GOT1. (b) Curve showing the variation of the net charge of WT GOT1 with pH, calculated using the Henderson-Hasselbalch equation. (c) Charge composition analysis of IDR1. (d) Curve showing the variation of the net charge of IDR1 with pH, calculated using the Henderson-Hasselbalch equation.

**4. Synthetic Methods and Scheme**

All reagents and anhydrous solvents were of commercial grade and used as received. Compound reactions were monitored under UV light at 254 nm or 365 nm. Compounds were purified by column chromatography, with progress monitored by thin-layer chromatography (TLC). Isolated products were characterized by ¹H-NMR and ¹³C-NMR spectroscopy in chloroform-d (CDCl₃) solvent.

Synthesis of Compound 1: Dissolve p-Bromobenzaldehyde (1g, 9.42 mmol) in dichloromethane and stir continuously until completely dissolved. Subsequently, add 2-methylpyrrole (906.88 μL, 18.84 mmol) to the dichloromethane solution (150 mL). Stir at room temperature on a magnetic stirrer at 450 rpm. Add 3 drops of trifluoroacetic acid as catalyst. React overnight. Add 1 equivalent of tetrachlorobenzoquinone as oxidant and react for 5 hours. Using a constant-pressure dropping funnel, add 10 mL of triethylamine dropwise. After 30 minutes, add 10 mL of boron trifluoride in diethyl ether dropwise. React overnight. Monitor reaction progress via thin-layer chromatography (TLC) by spotting the plate the following day. After reaction completion, transfer the flask contents to a separatory funnel. Add 50 mL DCM and 100 mL water to the funnel, mix, and extract the liquid phase into a conical flask. Dry the filtrate over anhydrous sodium sulfate and filter. Concentrate the mixture under reduced pressure. Purify via TLC using a petroleum ether: ethyl acetate solvent system (5:1, v/v) to obtain an orange solid compound 1 (890 mg, 89% yield).

Synthesis of Target Molecules: Adjust the nitrogen gas flow rate. Add magnetic stir bars to a 100 mL round-bottom flask. Weigh 1 mol (150 mg) of the purified product from the first reaction step. dissolve in 15 mL toluene solution. Add 3 mL DMF to dissolve 7 eq pyridoxal phosphate. Stir on magnetic stirrer. Rapidly add 1.34 eq p-toluenesulfonic acid and 150 μL piperidine to the reaction system. Install oil-water separator and condenser. Stir at 300 rpm, at 110°C overnight. Remove magnetic stir bar post-reaction. Evaporate to dryness under vacuum to remove toluene. Extract DMF from reaction mixture using saturated NaCl solution and ethyl acetate. Weigh 2 g silica gel powder and evaporate to dryness under vacuum. Purify by TLC using petroleum ether:ethyl acetate solvent system (6:1, v/v) to obtain the red target compound.
**5. NMR Spectra of Compounds**

**^1^H NMR spectrum of Compound 1**

**^13^C NMR spectrum of Compound 1**

**^1^H NMR spectrum of BDP-PLP**

**^13^C NMR spectrum of BDP-PLP**

**6. High resolution mass spectrometry (HRMS) of the compounds**

**HRMS of BDP-PLP**
